# *Neisseria gonorrhoeae* Coinfection during *Chlamydia muridarum* Genital Latency Does Not Modulate Murine Vaginal Bacterial Shedding

**DOI:** 10.1128/spectrum.04500-22

**Published:** 2023-04-11

**Authors:** Delia Onorini, Cory Ann Leonard, Regenia Phillips Campbell, Barbara Prähauser, Theresa Pesch, Robert V. Schoborg, Ann E. Jerse, Bernadetta Tarigan, Nicole Borel

**Affiliations:** a Institute of Veterinary Pathology, Vetsuisse Faculty, University of Zurich, Zurich, Switzerland; b Department of Medical Education, Center for Infectious Disease, Inflammation and Immunity, Quillen College of Medicine, East Tennessee State University, Johnson City, Tennessee, USA; c Department of Microbiology and Immunology, Uniformed Services University, Bethesda, Maryland, USA; d Department of Mathematics, Faculty of Science, University of Zurich, Zurich, Switzerland; Yale University

**Keywords:** *Chlamydia*, *Neisseria gonorrhoeae*, coinfection, mouse model

## Abstract

Chlamydia trachomatis and Neisseria gonorrhoeae are the most frequently reported agents of bacterial sexually transmitted disease worldwide. Nonetheless, C. trachomatis/N. gonorrhoeae coinfection remains understudied. C. trachomatis/N. gonorrhoeae coinfections are more common than expected by chance, suggesting C. trachomatis/N. gonorrhoeae interaction, and N. gonorrhoeae infection may reactivate genital chlamydial shedding in women with latent (quiescent) chlamydial infection. We hypothesized that N. gonorrhoeae would reactivate latent genital Chlamydia muridarum infection in mice. Two groups of C. muridarum-infected mice were allowed to transition into genital latency. One group was then vaginally inoculated with N. gonorrhoeae; a third group received N. gonorrhoeae alone. C. muridarum and N. gonorrhoeae vaginal shedding was measured over time in the coinfected and singly infected groups. Viable C. muridarum was absent from vaginal swabs but detected in rectal swabs, confirming C. muridarum genital latency and consistent with the intestinal tract as a C. muridarum reservoir. C. muridarum inclusions were observed in large intestinal, but not genital, tissues during latency. Oviduct dilation was associated with C. muridarum infection, as expected. Contradicting our hypothesis, N. gonorrhoeae coinfection did not reactivate latent C. muridarum vaginal shedding. In addition, latent C. muridarum infection did not modulate recovery of vaginal viable N. gonorrhoeae. Evidence for N. gonorrhoeae-dependent increased C. muridarum infectivity has thus not been demonstrated in murine coinfection, and the ability of C. muridarum coinfection to potentiate N. gonorrhoeae infectivity may depend on actively replicating vaginal C. muridarum. The proportion of mice with increased vaginal neutrophils (PMNs) was higher in N. gonorrhoeae-infected than in C. muridarum-infected mice, as expected, while that of C. muridarum/N. gonorrhoeae-coinfected mice was intermediate to the singly infected groups, suggesting latent C. muridarum murine infection may limit PMN response to subsequent N. gonorrhoeae infection.

**IMPORTANCE** Our work builds upon the limited understanding of C. muridarum/N. gonorrhoeae coinfection. Previously, N. gonorrhoeae infection of mice with acute (actively replicating) vaginal C. muridarum infection was shown to increase recovery of viable vaginal N. gonorrhoeae and vaginal PMNs, with no effect on C. muridarum vaginal shedding (R. A. Vonck et al., Infect Immun 79:1566–1577, 2011). It has also been shown that chlamydial infection of human and murine PMNs prevents normal PMN responses, including the response to N. gonorrhoeae (K. Rajeeve et al., Nat Microbiol 3:824–835, 2018). Our findings show no effect of latent genital C. muridarum infection on the recovery of viable N. gonorrhoeae, in contrast to the previously reported effect of acute C. muridarum infection, and suggesting that acute versus latent C. muridarum infection may have distinct effects on PMN function in mice. Together, these studies to date provide evidence that Chlamydia/N. gonorrhoeae synergistic interactions may depend on the presence of replicating Chlamydia in the genital tract, while chlamydial effects on vaginal PMNs may extend beyond acute infection.

## INTRODUCTION

Chlamydia trachomatis and Neisseria gonorrhoeae are the agents of most common bacterial sexually transmitted infections (STIs) worldwide, with 129 million new cases of chlamydia and 82 million new cases of gonorrhea reported in 2020 (1). These obligate human pathogens cause cervicitis in women and urethritis in men, with frequent asymptomatic and extragenital infection. Both can ascend to the upper genital tract, leading to pelvic inflammatory disease (PID), chronic abdominal pain, infertility, and ectopic pregnancy (2). C. trachomatis treatment failure is common (3, 4), progressive N. gonorrhoeae antimicrobial resistance is widespread (5), and no vaccines are available, despite decades of effort (6). The development of new C. trachomatis and N. gonorrhoeae therapies and vaccines is of critical public health concern.

Obligate intracellular C. trachomatis alternates between infectious elementary bodies (EBs) and replicative reticulate bodies (RBs). Inside host cells, EBs reside in membrane-bound inclusions and differentiate into RBs. After replication, RBs differentiate again into EBs and are released (7). Chlamydia induces innate and adaptive immune responses that may progress for months to years. The inflammatory response, important for infection resolution, also contributes to tissue damage and disease (8). Early in chlamydial infection, rapid neutrophil (PMN) influx is not sufficient to clear, but may limit, infection (9). C. trachomatis can survive inside PMNs, limiting PMN function (10). N. gonorrhoeae adheres to mucosal epithelia, forming microcolonies, and is capable of invasion and transcytosis, which can lead to disseminated infection (11). N. gonorrhoeae also recruits PMNs to the site of infection and can survive within PMNs. N. gonorrhoeae modulation of PMN function may also serve to facilitate N. gonorrhoeae growth and transmission (11–13).

C. trachomatis/N. gonorrhoeae coinfection may increase transmissibility and long-term complications. High rates of C. trachomatis coinfection occurred among young women with N. gonorrhoeae (14), and N. gonorrhoeae shedding was higher in C. trachomatis-coinfected women than in those with only N. gonorrhoeae (15). N. gonorrhoeae coinfection was a risk factor for recurring female C. trachomatis infection (16), which is associated with PID and reproductive complications (17). Notably, N. gonorrhoeae infection may reactivate latent (e.g., inapparent, undetected) C. trachomatis infection. Female contacts of men with C. trachomatis-negative, N. gonorrhoeae-positive urethritis had twice the chlamydial infection incidence of female contacts of men with C. trachomatis-negative, N. gonorrhoeae-negative urethritis (18). Recurring C. trachomatis infection with the same chlamydial serovar was also associated with N. gonorrhoeae coinfection (19). Similarly, 22% of female sex partners of N. gonorrhoeae-only infected men were C. trachomatis infected, further supporting that coinfection with N. gonorrhoeae may reactivate latent female C. trachomatis infection (20).

C. trachomatis/N. gonorrhoeae pathogenesis studies have focused largely on single pathogen infections (21–24). Chlamydia muridarum murine genital tract infection, used to model infection in women, induces inflammatory response and genital pathology similar to human disease (25). Experimental N. gonorrhoeae infection of estradiol-treated BALB/c mice is characterized by cervicovaginal infection with inflammatory PMN infiltration (26). The sole C. muridarum/N. gonorrhoeae coinfection mouse model showed higher viable N. gonorrhoeae vaginal load and vaginal PMNs in C. muridarum/N. gonorrhoeae-coinfected mice compared to N. gonorrhoeae-only infected mice (27). It was later shown that C. trachomatis infection of *ex vivo* human PMNs reduced subsequent N. gonorrhoeae-induced NET formation while increasing N. gonorrhoeae survival in PMNs (10). More recently, in a coinfection tissue culture model, we showed that N. gonorrhoeae elicited antichlamydial effects that were consistent with epithelial host cell sphingolipid depletion, suggesting C. trachomatis/N. gonorrhoeae interaction via host cell factors (28). These three studies, to our knowledge, are the only published experimental Chlamydia/N. gonorrhoeae coinfection studies. Although providing evidence of Chlamydia/N. gonorrhoeae interactions, they did not evaluate latent chlamydial infection.

We aimed here to evaluate the effect of N. gonorrhoeae coinfection specifically on latent C. muridarum infection. We hypothesized N. gonorrhoeae coinfection would reactivate latent genital chlamydial infection. We show (i) that N. gonorrhoeae does not reactivate C. muridarum from genital latency; (ii) that latent genital chlamydial infection, unlike acute genital chlamydial infection (27), does not increase the recovery of viable vaginal N. gonorrhoeae; and (iii) that C. muridarum infection may limit subsequent N. gonorrhoeae-dependent luminal vaginal PMN increase, consistent with chlamydial inhibition of PMN function (10). Our findings, together with previous studies, suggest differing effects of acute versus latent chlamydial infection on subsequent murine N. gonorrhoeae infection. Continued evaluation of Chlamydia/N. gonorrhoeae interaction, and how such coinfection may impact the host, is important to inform improved interventions for chlamydia and gonorrhea.

## RESULTS

### Study design and model characteristics.

**FIG 1:**
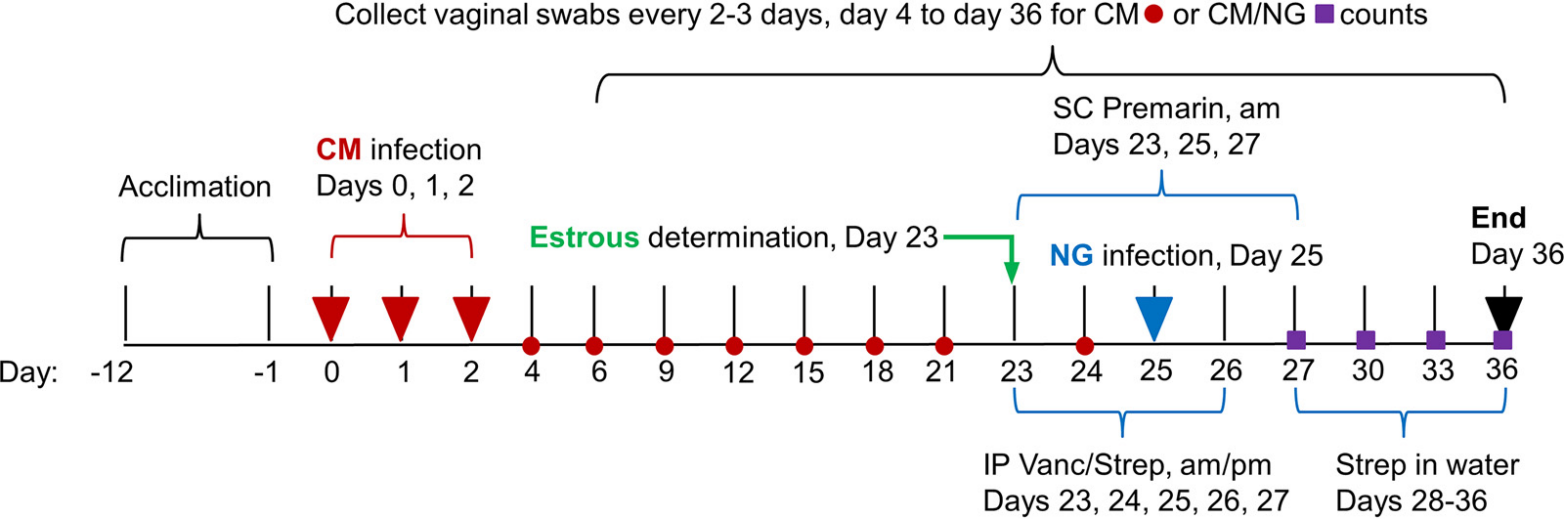
Experimental design. Mice were vaginally inoculated with C. muridarum (labeled “CM” in the figure) daily for three consecutive days. Viable C. muridarum shedding and recovery of viable N. gonorrhoeae (labeled “NG” in the figure) were monitored by regular vaginal swabbing, every 2 to 3 days, for the duration of the experiment. During chlamydial latency, vaginal smears were collected to determine estrous cycle stage, and selected mice (anestrus and diestrus stage) were treated with subcutaneous (labeled “SC” in the figure) injections of Premarin and intraperitoneal (labeled “IP” in the figure) injections of antibiotics (vancomycin hydrochloride and streptomycin sulfate [Vanc/Strep]) for 4 days, as shown. Two days after initiation of Premarin/antibiotics, mice were vaginally inoculated once with N. gonorrhoeae. After the cessation of antibiotic injections, Strep was supplied in drinking water. Mice that were not selected for Premarin/antibiotics treatment were sacrificed at day 23 (early sacrifice), and all other mice were sacrificed at day 36 (late sacrifice). At sacrifice, rectal swabs were collected for viable C. muridarum enumeration, and necropsy was performed to evaluate gross genital tract pathology; genital and intestinal tracts tissues were collected for evaluation of pathology.

Previously, acute C. muridarum genital infection increased recovery of viable N. gonorrhoeae and the influx of vaginal PMNs when N. gonorrhoeae infection was initiated 2 to 8 days after C. muridarum infection (27). Given that inapparent female C. trachomatis infection may be reactivated by N. gonorrhoeae (18–20), we hypothesized that murine N. gonorrhoeae coinfection during chlamydial genital latency would reactivate latent C. muridarum infection. We delayed N. gonorrhoeae infection until 23 days after the final C. muridarum inoculation (Fig. 1) to coincide with chlamydial genital latency, as characterized by undetectable C. muridarum vaginal shedding, but continued intestinal infection. Mice in estrus have limited susceptibility to C. muridarum infection via vaginal inoculation (29), and inoculation for three consecutive days ensures mice are exposed to C. muridarum during the luteal stage of the 4- to 5-day cycle. Inoculation with 10^5^ to 10^7^ inclusion forming units (IFU) of C. muridarum typically results in peak infection around 10^4^ to 10^5^ IFU/vaginal swab, lasting 15 to 21 days (30). In a pilot study (*n* = 10 mice), 3 days of vaginal inoculation with 10^5^ IFU/day resulted in limited infection (50% of mice), with 10^1^ to 10^4^ maximum IFU/swab detectable for only ~1 week (data not shown). Therefore, we used an inoculum dose of 10^6^ C. muridarum IFU.

We monitored vaginal C. muridarum shedding to confirm decline to genital latency. To confirm mice harbored viable C. muridarum and infection had not been cleared, we monitored rectal shedding of C. muridarum, since the intestinal tract may serve as a C. muridarum reservoir for recrudescent vaginal infection (31, 32). Mice were then evaluated for estrous stage, and those in anestrus or diestrus were treated with Premarin and antibiotics to facilitate robust, extended N. gonorrhoeae infection (23, 27). After vaginal N. gonorrhoeae coinfection, we monitored vaginal viable C. muridarum shedding and recovery of viable N. gonorrhoeae to determine impact of coinfection, comparing singly infected (C. muridarum or N. gonorrhoeae) versus C. muridarum/N. gonorrhoeae-coinfected groups. We collected genital and intestinal tract tissues for pathology analyses and immunohistochemical (IHC) analyses for C. muridarum and N. gonorrhoeae in these tissues, and we used vaginal smears to enumerate vaginal luminal PMNs.

The study consisted of three independent experiments (10 to 40 mice each, 90 total mice; see File S1 in the supplemental material). Mice were divided into four groups: coinfected with C. muridarum and N. gonorrhoeae, infected with either pathogen alone (C. muridarum or N. gonorrhoeae) or control, mock-infected (UN). One mouse died spontaneously on day 1, prior to completion of C. muridarum infection, with no pathological findings, and was excluded from analyses. No other mice showed negative impact on well-being (see File S2). On day 23, mice not in anestrus or diestrus, and thus suboptimal for Premarin treatment (33), were sacrificed (“early sacrifice”). Mice in anestrus or diestrus remained in the study until the end of experiment and were Premarin/antibiotic treated, mock or N. gonorrhoeae inoculated, and sacrificed on day 36 (“late sacrifice”). Mice without detectable C. muridarum or N. gonorrhoeae shedding were excluded from vaginal bacterial shedding (but not pathology) analyses (see File S1 for the numbers of mice in each group).

In the first independent experiment (experiment 1, *n* = 10 mice; see File S1 and Table S1 in the supplemental material), we compared C. muridarum viable vaginal shedding (C. muridarum titer) and C. muridarum genome (Chlamydia qPCR, C. muridarum qPCR) positivity for all vaginal swabs collected (*n* = 106 swabs). Two mice were from the early sacrifice on day 23, coinciding with C. muridarum genital latency prior to subsequent N. gonorrhoeae infection (one mock infected and one C. muridarum infected); the eight remaining mice were sacrificed on day 36, at the end of experiment (four N. gonorrhoeae and four C. muridarum+N. gonorrhoeae). As expected, all swabs from mice not infected with C. muridarum were titer and qPCR negative. Swabs collected from C. muridarum-infected mice on days 4 to 21 (*n* = 31 swabs) were 45.2% C. muridarum titer and qPCR positive. Eleven swabs were determined to be positive by titer and qPCR, three swabs were positive by titer only, three swabs were positive by qPCR only. All swabs from days 24 to 36 were titer and qPCR negative. Given approximately equivalent C. muridarum titer and qPCR positivity, vaginal swab C. muridarum genomes were not monitored for the remainder of the study.

### Vaginal *N. gonorrhoeae* coinfection does not reactivate latent genital chlamydial shedding.

**FIG 2:**
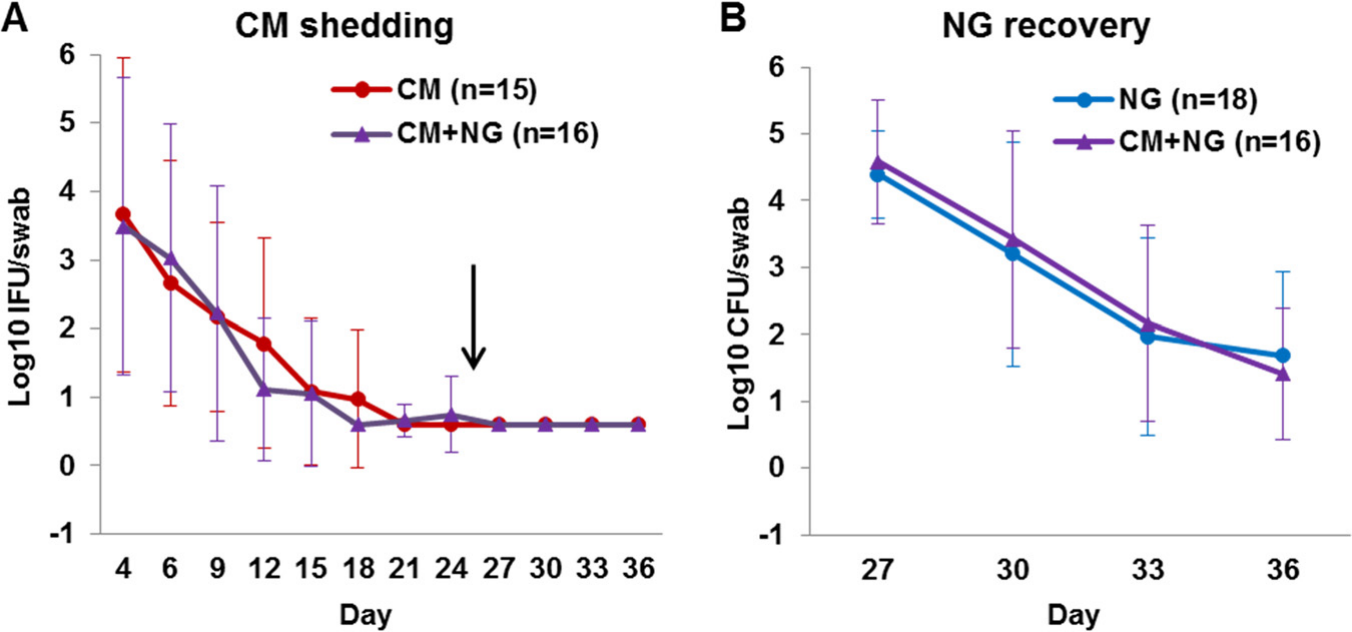
Vaginal viable C. muridarum (labeled “CM” in the figure) and N. gonorrhoeae (labeled “NG” in the figure). (A and B) Quantification of viable C. muridarum shedding was determined by infecting LLC cells (A), and quantification of viable N. gonorrhoeae recovery was determined by culturing on commercial agar plates selective for N. gonorrhoeae (B). The results are expressed as mean log_10_ IFU or CFU per vaginal swab ± the SD. The arrow in panel A indicates N. gonorrhoeae vaginal inoculation on day 25.

The primary outcome evaluated was the number of viable bacteria recovered from vaginal swabs. We hypothesized latent genital C. muridarum shedding would be reactivated by N. gonorrhoeae coinfection. Shedding was assessed by quantitative titer assay, and analyzed and presented as mean “C. muridarum shedding” in log_10_ C. muridarum IFU/swab (standard deviation [SD]) and mean “N. gonorrhoeae recovery” in log_10_ N. gonorrhoeae CFU/swab (SD) (Fig. 2). Until day 24 of the study, mice in the C. muridarum and C. muridarum+N. gonorrhoeae groups exhibited similar expected acute vaginal shedding. Shedding peaked on day 4 and became undetectable by day 24, indicating genital latency (Fig. 2A). The day 4 mean log_10_ (SD) IFU/swab for C. muridarum was 3.66 (2.29), and that for C. muridarum+N. gonorrhoeae was 3.49 (2.17); the result for both groups combined was 3.57 (2.17). The day 4 log_10_ IFU/swab ranges were as follows: C. muridarum = 0.6 to 7.06, C. muridarum+N. gonorrhoeae = 0.6 to 6.55, and all C. muridarum infected = 0.6 to 7.06.

Detection of C. muridarum vaginal shedding began primarily on day 4 (83.9%) and in fewer mice on day 6 (9.7%) or day 9 (6.5%) (Table 1). Mice had detectable C. muridarum shedding in one to six vaginal swabs of the twelve swabs collected. All mice ceased detectable shedding by day 24, including those excluded due to the lack of detectable N. gonorrhoeae vaginal shedding. No mice in the UN or N. gonorrhoeae groups had detectable viable C. muridarum vaginal shedding in any swabs (see Materials and Methods for the contamination check protocol). Thus, although C. muridarum vaginal shedding declined to genital latency as expected, in contradiction to our hypothesis, N. gonorrhoeae coinfection failed to cause resumed C. muridarum shedding in latently C. muridarum-infected mice (Fig. 2A). Critically, preliminary experiments showed that it is possible for C. muridarum vaginal shedding to be reactivated from an undetectable level upon immune suppression in our mouse model (BALB/c mice and C. muridarum Weiss strain; see File S3).

**TABLE 1:** Viable C. muridarum vaginal shedding

Infection	No. of positive mice/total no. of mice (%)								
	Day 4	Day 6	Day 9	Day 12	Day 15	Day 18	Day 21	Day 24	Days 27–36
C. muridarum	11/15 (73.3)	12/15 (80)	12/15 (80)	7/15 (46.7)	4/15 (26.7)	2/15 (13.3)	0/15	0/15	0/15
C. muridarum+N. gonorrhoeae	15/16 (93.8)	12/16 (75)	11/16 (68.8)	5/16 (31.3)	3/16 (18.8)	0/16	1/16 (6.3)	1/16 (6.3)	0/16
C. muridarum infected	26/31 (83.9)	24/31 (77.4)	23/31 (74.2)	12/31 (38.7)	7/31 (22.6)	2/31 (6.5)	1/31 (3.2)	1/31 (3.2)	0/31

### Latent *C. muridarum* genital infection is associated with rectal viable chlamydial shedding.

We evaluated viable C. muridarum rectal shedding in swabs collected during necropsy at early and late sacrifice. In addition, C. muridarum qPCR analysis of rectal swabs was performed for all C. muridarum-inoculated mice (Table 2; see also File S1). As expected, all early-sacrifice mock-infected mice and all late-sacrifice UN and N. gonorrhoeae-infected mice showed no detectable viable C. muridarum rectal shedding (see Materials and Methods for contamination check protocol).

**TABLE 2:** Mice with C. muridarum rectal carriage

Infection	C. muridarum titer		C. muridarum qPCR	
	No. of positive mice/total no. of mice (%)	Mean log_10_ (SD) C. muridarum IFU/rectal swab	No. of positive mice/total no. of mice (%)	Mean log_10_ (SD) C. muridarum genomes/rectal swab
Early sacrifice				
Mock infected	0/12		0/12	
C. muridarum infected	13/13 (100)	2.89 (0.62)	13/13 (100)	3.50 (0.75)
Late sacrifice				
Mock infected	0/4		0/4	
C. muridarum	4/15 (26.7)	0.84 (0.45)	9/15 (60)	1.79 (1.10)
N. gonorrhoeae	0/24		0/24	
C. muridarum+N. gonorrhoeae	5/21 (23.8)	0.88 (0.64)	8/21 (38.1)	1.34 (1.02)

All C. muridarum-infected early-sacrifice mice were both C. muridarum rectal titer and rectal qPCR positive 2 days prior to N. gonorrhoeae infection (day 25), indicating that the mice had not cleared C. muridarum infection (Table 2). Late-sacrifice mice were analyzed whether or not they had detectable C. muridarum or N. gonorrhoeae vaginal shedding, and rectal C. muridarum positivity ranged from approximately 20 to 60%; no mice with undetectable C. muridarum (*n* = 1) or N. gonorrhoeae (*n* = 4) vaginal shedding were C. muridarum rectal titer or qPCR positive. There was no difference in mean C. muridarum IFU/rectal swab or in mean C. muridarum genomes/rectal swab between C. muridarum and C. muridarum+N. gonorrhoeae groups, nor were the proportions of titer- or qPCR-positive mice significantly different for the C. muridarum versus the C. muridarum+N. gonorrhoeae groups. Thus, we found no indication that N. gonorrhoeae coinfection modulates latent rectal C. muridarum shedding.

### Latent chlamydial genital infection does not modulate the recovery of viable *N. gonorrhoeae*.

On days 27 to 36, N. gonorrhoeae-infected and C. muridarum+N. gonorrhoeae-infected mice exhibited robust and prolonged vaginal N. gonorrhoeae colonization similar to that previously described (27). Viable N. gonorrhoeae recovery peaked on day 27, followed by gradual decline until day 36. Specifically, the mean log_10_ (SD) CFU/swab on day 27 for N. gonorrhoeae was 4.39 (0.65), and that for C. muridarum+N. gonorrhoeae was 4.58 (0.92); the mean log_10_ (SD) CFU/swab on day 36 for N. gonorrhoeae was 1.68 (1.25), and that for C. muridarum+N. gonorrhoeae was 1.41 (0.98). Individual day 27 and day 36 log_10_ (SD) CFU/swab ranges were N. gonorrhoeae = 3.32 to 5.67 and C. muridarum+N. gonorrhoeae = 3.08 to 5.71 and were N. gonorrhoeae = 2.39 to 4.12 and C. muridarum+N. gonorrhoeae = 1.29 to 4.06, respectively (Fig. 2B).

N. gonorrhoeae and C. muridarum+N. gonorrhoeae groups had detectable N. gonorrhoeae shedding in one to four of the four vaginal swabs collected after N. gonorrhoeae infection; at least 25% of mice in both groups had detectable shedding on day 36 (Table 3). Viable N. gonorrhoeae vaginal shedding in the N. gonorrhoeae and C. muridarum+N. gonorrhoeae groups was detected starting on day 27. No mice in the UN or C. muridarum groups showed detectable viable N. gonorrhoeae shedding in any swabs (see Materials and Methods for the contamination check protocol). Mean viable N. gonorrhoeae vaginal shedding did not differ significantly between the N. gonorrhoeae and C. muridarum+N. gonorrhoeae groups (Fig. 2B). Thus, in contrast to the previous report that acute genital C. muridarum infection enhances vaginal shedding of live N. gonorrhoeae during subsequent N. gonorrhoeae coinfection (27), we found no evidence that latent genital C. muridarum infection can exert a similar effect on N. gonorrhoeae infection in this mouse model.

**TABLE 3:** Mice with viable N. gonorrhoeae vaginal shedding

Infection	No. of positive mice/total no. of mice (%)			
	Day 27	Day 30	Day 33	Day 36
N. gonorrhoeae	18/18 (100)	13/18 (72.2)	7/18 (38.9)	5/18 (27.8)
C. muridarum+N. gonorrhoeae	16/16 (100)	12/16 (75)	7/16 (43.8)	4/16 (25)

### Genital tract *N. gonorrhoeae*, but not *C. muridarum*, was detected during chlamydial genital latency.

For all fixed tissue analyses, early-sacrifice mice (i.e., sacrificed *before* N. gonorrhoeae inoculation) were evaluated as two groups: mock infected and C. muridarum infected. Late-sacrifice mice, i.e., sacrificed *after* N. gonorrhoeae or mock inoculation on day 25, were analyzed as UN, C. muridarum, N. gonorrhoeae, and C. muridarum+N. gonorrhoeae groups. By IHC, C. muridarum inclusions were not observed in the genital tracts of any mice, including those determined to be positive by Chlamydia (C. muridarum) qPCR evaluation of genital tissue (see Table S2). Genital tract tissue determined to be negative by C. muridarum qPCR was not evaluated by C. muridarum IHC. In addition, of ten C. muridarum mice from a pilot study with 10^5^ IFU inocula (data not shown), two, both early sacrifice, were genital tract C. muridarum qPCR positive but C. muridarum IHC negative.

**FIG 3:**
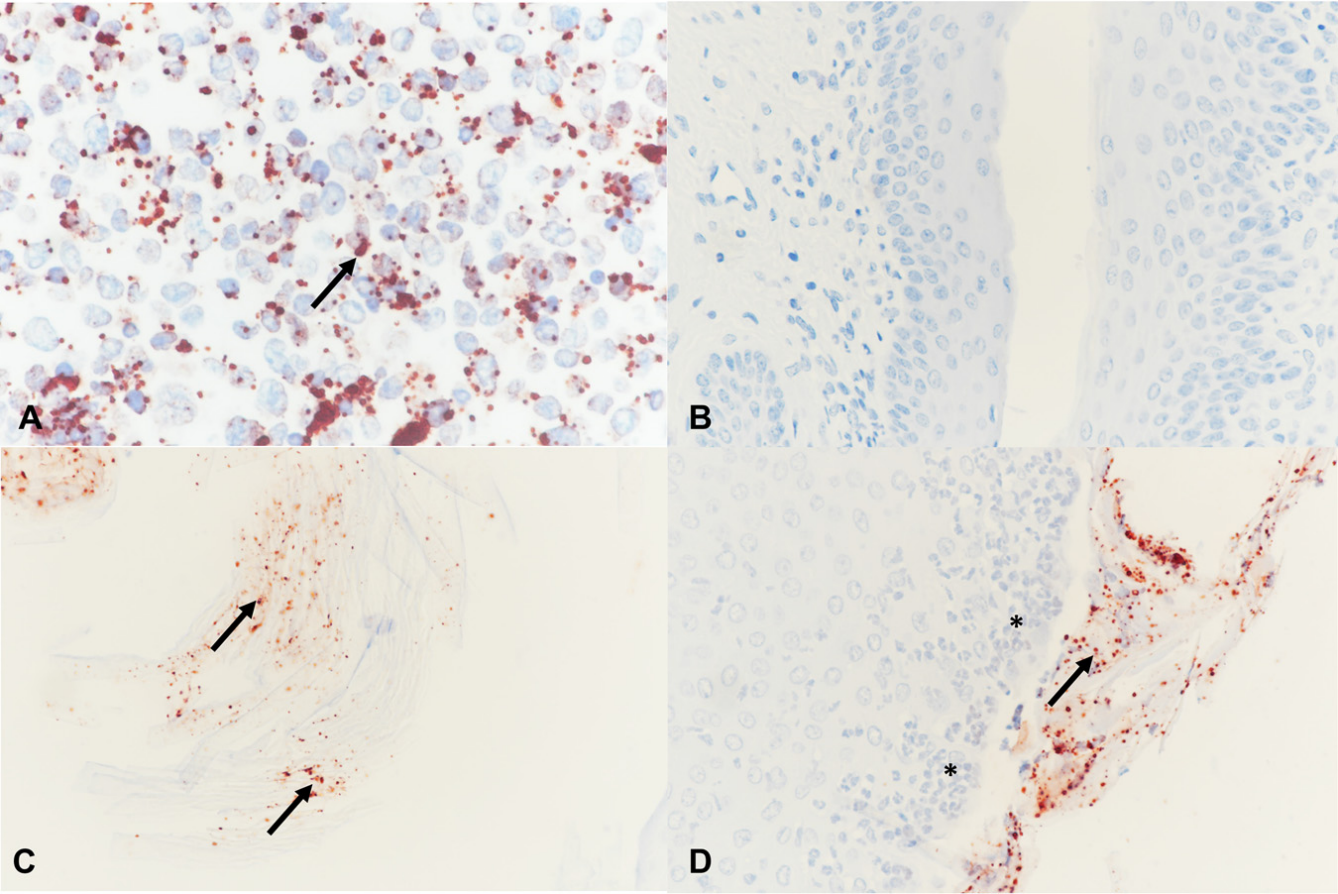
*Neisseria* (N. gonorrhoeae) immunohistochemistry (IHC) was performed in the positive-control cell pellet (A) and in mouse vagina (B to D) with red positively labeled extracellular N. gonorrhoeae (arrows). (A) Cell pellet infected with N. gonorrhoeae. (B) Vagina negative IHC, late-sacrifice N. gonorrhoeae group. (C) Luminal N. gonorrhoeae attached to cornified epithelial cells, late-sacrifice C. muridarum+N. gonorrhoeae group. (D) N. gonorrhoeae attached to superficial layers of the vaginal epithelium, late-sacrifice N. gonorrhoeae group. The vaginal epithelium is infiltrated with PMNs (asterisks). Magnification, ×400.

*Neisseria* (N. gonorrhoeae) IHC was performed on 77 mice, including all N. gonorrhoeae-inoculated (e.g., N. gonorrhoeae and C. muridarum+N. gonorrhoeae late-sacrifice) mice for which vaginal tissue was available for analysis (*n* = 42; see Table S3). As expected, the genital tracts of mice not N. gonorrhoeae inoculated were N. gonorrhoeae IHC negative. Approximately 20 to 40% of N. gonorrhoeae-inoculated late-sacrifice mice were N. gonorrhoeae IHC positive, and the proportion of positive mice did not differ between the two groups. Positive N. gonorrhoeae labeling was present in the vagina and not other genital tract anatomical sites, as expected, since human transferrin is required for ascended N. gonorrhoeae infection in mice (34). N. gonorrhoeae vaginal positivity was primarily luminal, attached to the surface epithelium or to detached/luminal cornified epithelial cells (Fig. 3).

### *Chlamydia* infection of the large intestine during latent genital chlamydial infection.

**FIG 4:**
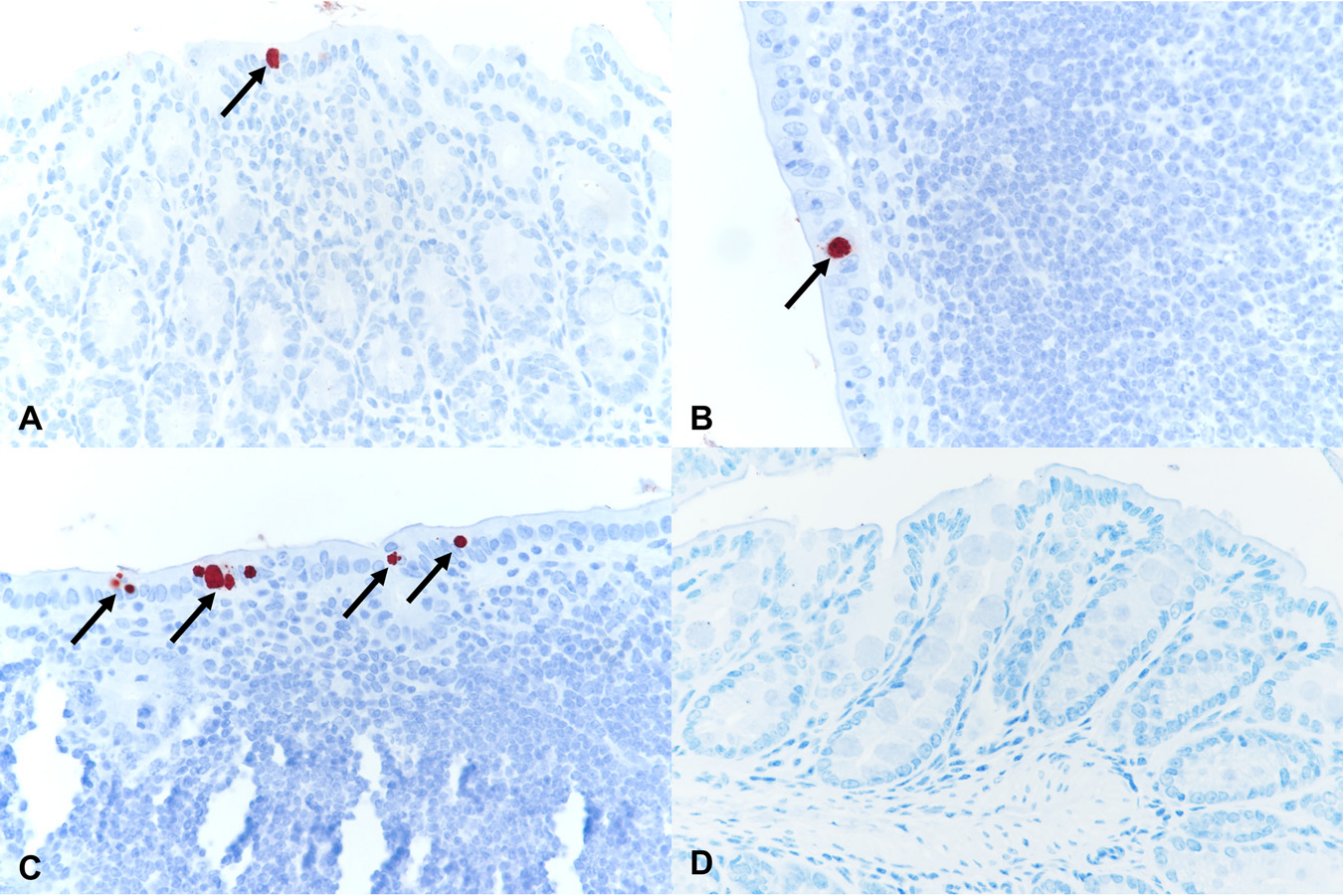
Chlamydiaceae immunohistochemistry in mouse intestines with red positively labeled intracytoplasmic chlamydial inclusions (arrows) in the intestinal epithelium of the early-sacrifice C. muridarum-infected group. (A) Cecum, early-sacrifice C. muridarum-infected group. (B) Colon, early-sacrifice C. muridarum-infected group. (C) Rectum, early-sacrifice C. muridarum-infected group. (D) Negative rectum, late-sacrifice C. muridarum+N. gonorrhoeae group. Magnification, ×400.

Given the observed rectal swab C. muridarum positivity, duodenum, jejunum, ileum, cecum, colon, and rectum tissues of C. muridarum-infected early-sacrifice and C. muridarum and C. muridarum+N. gonorrhoeae late-sacrifice mice with C. muridarum qPCR-positive intestinal tissue (*n* = 16; see Table S4) were investigated by C. muridarum IHC. If a single C. muridarum inclusion was observed, the mouse was considered C. muridarum IHC positive. Of the C. muridarum qPCR-positive mice, >50% (*n* = 9), all early-sacrifice C. muridarum-infected mice, were IHC positive (Table 4). Inclusions per intestinal cross section ranged from 1 up to 10 to 15 (data not shown). C. muridarum inclusions were observed in the intestinal epithelium of the cecum, colon, and/or rectum (large intestine), but not in the duodenum, jejunum, or ileum (small intestine) (Table 4). Examples of positive C. muridarum IHC are shown in Fig. 4.

**TABLE 4:** Positive Chlamydia intestinal tract immunolabeling per anatomical localization

Anatomical localization	Positive immunolabeling (no.)
Cecum only	2
Colon only	1
Rectum only	2
Cecum + colon	2
Cecum + rectum	1
Colon + rectum	1
Total	9

### Gross pathology and histopathology of the genital tract.

Gross genital tract pathology of all mice was unremarkable (genital tracts were similar in appearance across all groups, including uninfected controls, and showed no marked swelling, redness, or other abnormalities). For all mice, one to three block sections per fixed genital tissue block were prepared to help facilitate assessment of the full genital tract (longitudinal section). The ovary, missing from sections of five mice, had a similar appearance across all experimental groups and was not further studied. The uterus, available but similar in appearance in all mice, showing only estrus cycle-dependent lumen dilation (mild, moderate, or severe), was not further evaluated (data not shown).

**FIG 5:**
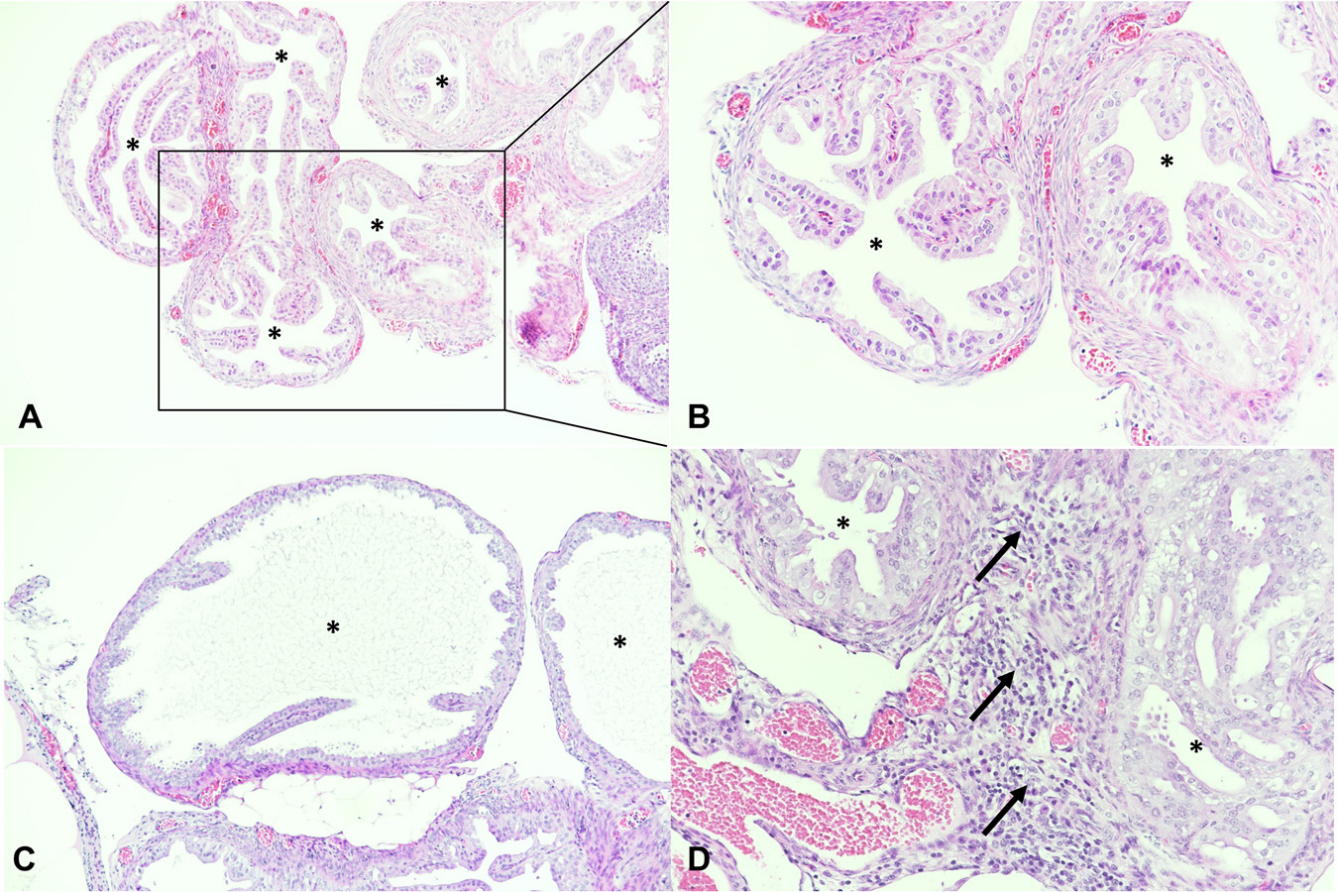
H&E staining of oviducts (asterisks). (A and B) Normal oviduct cross sections, late-sacrifice N. gonorrhoeae group. Magnifications, ×100 (A) and ×200 (B, see inset A). (C) Dilated oviducts (asterisk), late-sacrifice C. muridarum group. Magnification, ×100. (D) Peroviductal inflammation (arrows), mostly lymphocytes, early-sacrifice C. muridarum-infected group. Magnification, ×200.

At least one oviduct cross section was present in at least one genital block section for all except two mice, which were excluded from analysis. Oviduct cross sections/mouse ranged from 1 to 17 (mean = 6.4, SD = 4.0), and dilation was typically observed in 1 to 2 cross sections/mouse (Fig. 5). The proportion of mice with dilation ranged from approximately 16 to 40%, and the proportion of mice with inflammatory infiltrates ranged from approximately 4 to 26% across all groups. Inflammatory infiltrates were localized periductal, mostly mild, and focal and consisted of a mixture of inflammatory cells such as PMNs, macrophages, lymphocytes, or plasma cells (Fig. 5). Perioviductal inflammation was associated with oviduct dilation in six mice. Among the early-sacrifice mice, 20% with oviduct dilation also had periductal inflammation, while 33.3% with periductal inflammation also had oviduct dilation. Among the late-sacrifice mice, 31.25% with oviduct dilation also had periductal inflammation, while 62.5% with periductal inflammation also had oviduct dilation. C. muridarum-induced hydrosalpinx was not observed.

**FIG 6:**
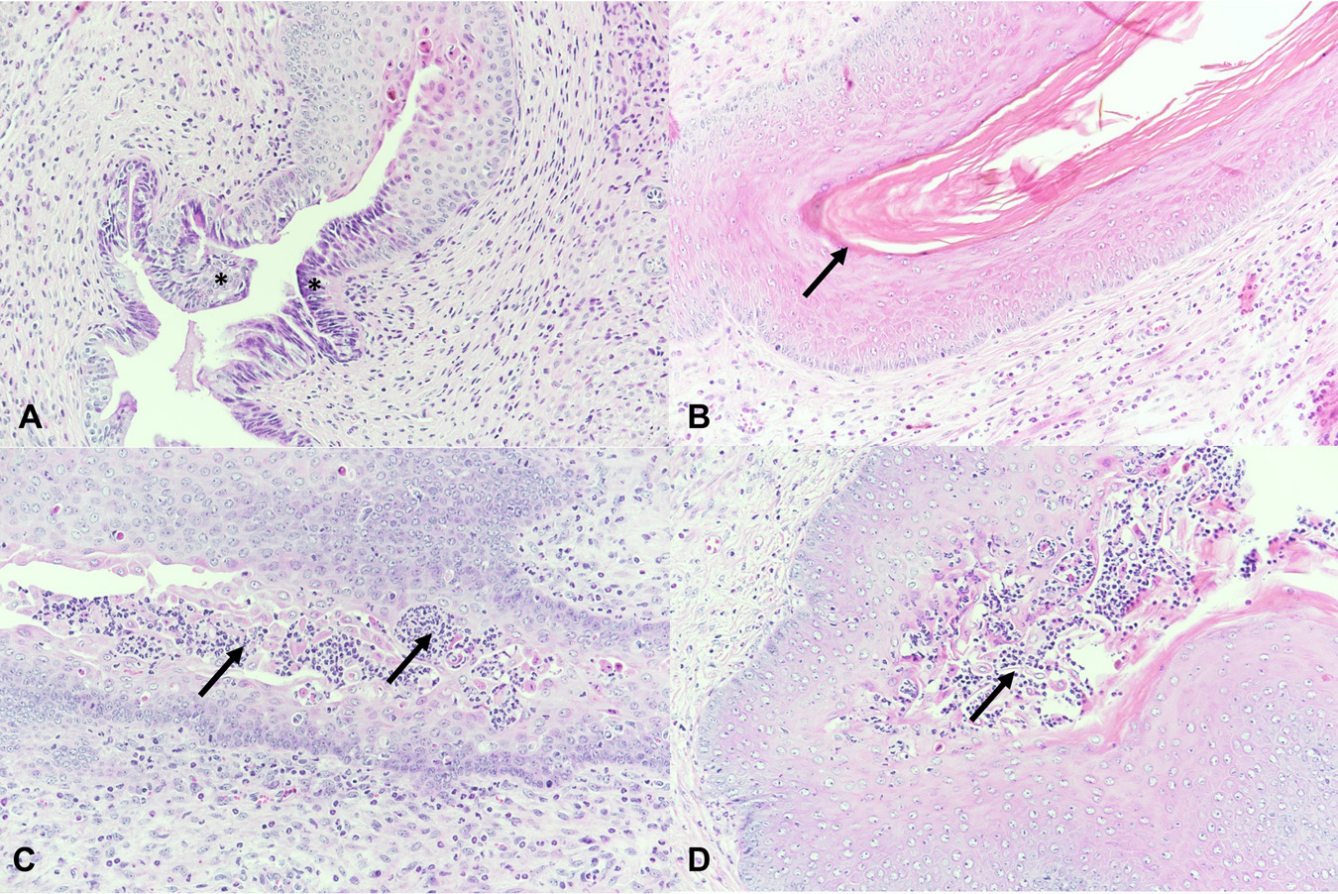
H&E staining of cervix and vagina. (A) Normal cervix with transition from columnar to stratified epithelium (asterisks); late-sacrifice N. gonorrhoeae group. (B) Normal vagina lined by keratinized squamous epithelium (arrow); late-sacrifice N. gonorrhoeae group. (C) Cervix, severe infiltration with PMNs (arrows), late-sacrifice C. muridarum group. (D) Vagina, severe infiltration with PMNs (arrows), late-sacrifice N. gonorrhoeae group. Magnification, ×200.

The cervix, present in 85 mice, was lined by columnar epithelium transitioning to stratified epithelium. Cervical PMN luminal accumulation and/or intraepithelial infiltration, was graded as none, mild, moderate, or severe. The proportion of mice with cervical PMN accumulation/infiltration ranged from approximately 27 to 41% across all groups (see Table S6 in the supplemental material). PMN intraepithelial infiltration (*n* = 17) was more common than combined luminal/intraepithelial accumulation/infiltration (*n* = 10) or luminal accumulation (*n* = 1). Accumulation/infiltration ranged from mild (*n* = 13) to moderate (*n* = 6) to severe (*n* = 9) and did not differ between luminal and intraepithelial sites when present at both (Fig. 6).

The vagina, present in 84 mice, was mostly lined by a keratinized squamous epithelium depending on estrus stage (primarily estrus-like). Vaginal PMN luminal accumulation and/or intraepithelial infiltration, was graded as none, mild, moderate, or severe. The proportion of mice with vaginal PMN accumulation/infiltration ranged from approximately 40 to 75% across all groups (see Table S7). PMN intraepithelial infiltration (*n* = 31) was more common than combined luminal/intraepithelial accumulation/infiltration (*n* = 16) or luminal accumulation (*n* = 1). Accumulation/infiltration ranged from mild (*n* = 21) to moderate (*n* = 16) to severe (*n* = 11) and did not differ between luminal and intraepithelial sites when present at both (Fig. 6).

In 20 mice, cervical and vaginal PMN infiltration was present simultaneously. In early-sacrifice mice, 100% (of 9) of mice with cervical inflammation had concomitant vaginal inflammation, while 9/15 (60%) with vaginal inflammation had cervical inflammation. In late-sacrifice mice, 11/19 mice (57.9%) with cervical inflammation had concomitant vaginal inflammation, while 11/32 (34.4%) had cervical inflammation.

Statistical analysis was performed on histopathology data for early-sacrifice mice, comparing mock-infected and C. muridarum-infected groups, and late-sacrifice mice, comparing UN, C. muridarum, N. gonorrhoeae, and C. muridarum+N. gonorrhoeae groups, minus any mice with missing relevant sections. Five pathology outcomes were considered: oviduct dilation ratio (dilated/total oviduct cross sections), oviduct dilation score (none, mild, or moderate), and oviduct inflammation (yes versus no), as well as cervical and vaginal infiltration with PMNs graded from 0 to 3 (none, mild, moderate, or severe). No outcomes were statistically different between groups (see Materials and Methods for details of statistical analyses).

### Latent genital *C. muridarum* infection may limit subsequent *N. gonorrhoeae*-dependent increases in vaginal luminal PMNs.

The proportion of mice with vaginal tissue inflammation was not increased in coinfected versus single-infected mice (see Table S7). Therefore, we evaluated vaginal luminal PMN numbers by microscopic analysis of vaginal smears (*n* = 64 late-sacrifice mice). Vaginal smears were categorized as having either no/rare PMNs (consistent with estradiol/estrogen-induced estrus; negative for increased vaginal luminal PMNs) or few/more PMNs (consistent with PMN influx into the vaginal lumen; positive for increased vaginal luminal PMNs). The relative proportions of mice with few/more PMNs were ~25% for the UN and C. muridarum groups, 65% for the N. gonorrhoeae group, and <50% for the C. muridarum+N. gonorrhoeae group. For the three infected groups, differences between proportion of positivity approached statistical significance, as determined by Fisher exact test (*P* = 0.05332). Pairwise comparisons, however, approached significant differences only for the N. gonorrhoeae and C. muridarum groups (*P* = 0.06743), while remaining pairwise comparisons yielded *P* values of >0.3. These observed trends (in vaginal tissues and vaginal smears) suggest that C. muridarum/N. gonorrhoeae-coinfected mice have a proportion of increased vaginal luminal PMNs intermediate to that of singly infected mice.

## DISCUSSION

C. trachomatis/N. gonorrhoeae interaction may increase host susceptibility and/or transmissibility, the latter potentially due to a C. trachomatis-mediated increase in N. gonorrhoeae bacterial load (15, 27). N. gonorrhoeae may increase incident C. trachomatis infection (35) and/or reactivate latent female genital chlamydial shedding (18–20), and C. trachomatis/N. gonorrhoeae coinfection may promote symptoms and negative health outcomes (35, 36). Three published coinfection studies exist to date, two providing *in vivo* and *ex vivo* evidence that active Chlamydia infection may influence N. gonorrhoeae infection (10, 27), while our more recent study showed N. gonorrhoeae antichlamydial effects in an epithelial *in vitro* model (28). Novel models and continued study of C. trachomatis/N. gonorrhoeae coinfection, in the context of productive and latent Chlamydia infection, will improve understanding of C. trachomatis/N. gonorrhoeae interaction.

Mice vaginally infected with C. muridarum (previously referred to as C. trachomatis MoPn, [37]) naturally progress to quiescent genital latency, and detectable vaginal C. muridarum shedding can be reactivated by pharmacological immune suppression (38; see also File S3 in the supplemental material). We hypothesized that N. gonorrhoeae coinfection would reactivate vaginal shedding in mice with latent genital C. muridarum infection, considering such reactivation may occur in women (18–20), and evaluated this hypothesis by modifying the sole existing mouse C. muridarum/N. gonorrhoeae coinfection model (27). Mice showed expected early peak viable vaginal C. muridarum shedding waning to undetectable levels, characteristic of genital latency (30, 38), and mice sacrificed at this time were 100% positive for viable rectal C. muridarum carriage. Thus, prior to N. gonorrhoeae infection, mice were not entirely cured of C. muridarum infection, having potential for reactivation of C. muridarum vaginal shedding, consistent with the intestine as a C. muridarum reservoir in mice (31, 32). Resumed vaginal C. muridarum shedding was not detected upon N. gonorrhoeae coinfection, however, contradicting our hypothesis.

Previous studies showed vaginal C. muridarum inoculation resulted in robust genital and intestinal infection (39), while intragastric or rectal C. muridarum inoculation resulted in intestinal infection, without autoinoculation of the genital tract, even after 70 days (40). Vaginal inoculation with C. muridarum attenuated for upper genital tract pathology, followed by intragastric wild-type C. muridarum coinoculation, also did not result in C. muridarum spread to the genital tract (41). It remains possible that C. muridarum intestinal infection could serve as a source for reactivation, though undetectable C. muridarum genital infection and/or genital persistent/aberrant chlamydial forms (42) may be the source of reactivated latent C. muridarum (38).

In our study, C. muridarum singly infected and C. muridarum/N. gonorrhoeae-coinfected groups showed similar C. muridarum infection characteristics prior to N. gonorrhoeae infection, largely precluding confounding differences in early C. muridarum infection. However, a major limitation of our study is the requirement for estradiol/estrogen treatment to promote N. gonorrhoeae infection, given that this induced state of estrus is likely unfavorable for C. muridarum genital infection. Similar estradiol treatment in the original C. muridarum/N. gonorrhoeae coinfection model did not prevent acute vaginal C. muridarum infection and viable shedding, nor was acute C. muridarum shedding increased by N. gonorrhoeae coinfection (27). However, we cannot rule out estrogen-limited C. muridarum susceptibility playing a role in the failure of N. gonorrhoeae coinfection to elicit reactivation of viable vaginal C. muridarum shedding in mice with latent genital C. muridarum infection.

In contrast to acute C. muridarum genital infection increasing subsequent recovery of viable vaginal N. gonorrhoeae (27), we did not observe a similar N. gonorrhoeae-enhancing effect for latent genital C. muridarum infection. This may indicate, since there is no detectable C. muridarum vaginal shedding in genital C. muridarum latency, that actively replicating C. muridarum must be present in the murine vagina to elicit the effect on vaginal N. gonorrhoeae. In our recent *in vitro* study of genital epithelial cell coinfection, N. gonorrhoeae limited chlamydial development/infectivity in a manner consistent with host sphingolipid depletion; this effect also required live N. gonorrhoeae (killed N. gonorrhoeae and lysates failed to have an effect), providing an example of N. gonorrhoeae/C. trachomatis interaction via host cell factors that requires concomitant viability of both bacteria (28). In addition, in *in vitro* coinfection, N. gonorrhoeae CFU/mL was not increased in C. trachomatis/N. gonorrhoeae-coinfected versus N. gonorrhoeae singly infected samples; however, only a single final CFU/mL measurement of nonadherent and noninvaded N. gonorrhoeae present in the culture medium was made at the experimental endpoint (28).

Previously, in acute murine C. muridarum genital infection, C. muridarum inclusions were found in the cervix and N. gonorrhoeae was found in the cervix and vagina 2 days after N. gonorrhoeae infection, when live vaginal shedding for both bacteria was ~3 log_10_ IFU or CFU/swab (27). In contrast, we observed N. gonorrhoeae only in the vagina, and no C. muridarum inclusions in the genital tract, at 11 days after N. gonorrhoeae infection and 34 days after C. muridarum infection, presumably due to increased time postinfection. Notably, acute C. muridarum genital infection was previously associated with increased levels MIP-2 and TNF-α (inflammatory mediators) and decreased transcription of two antimicrobial peptides (CRAMP and SLPI) prior to N. gonorrhoeae infection, the latter of which may explain acute C. muridarum-dependent enhancement of N. gonorrhoeae vaginal shedding (27). While we did not evaluate antimicrobial peptide transcription, it is possible they are important for the effect of acute genital C. muridarum infection on N. gonorrhoeae vaginal shedding, but are no longer modulated during C. muridarum genital latency.

Genital tract pathology in our progesterone-free coinfection model was milder than generally reported for progesterone-enhanced C. muridarum murine vaginal infection, which typically induces hydrosalpinx (43, 44). We assessed pathology 34 days after C. muridarum infection, and genital pathology measures are often made later, even up 80 days postinfection (43). In progesterone-assisted C. muridarum infection, hydrosalpinx can be observed by 35 days postinfection (45). Previously, progesterone-free C. muridarum vaginal infection in BALB/c mice also failed to show hydrosalpinx (46), so mouse strain and hormone-free C. muridarum infection may play a role. A large C. muridarum inoculum (10^5^ to 10^7^ IFU, similar to our inoculum of 10^6^) has been shown to result in *less* ascending infection and hydrosalpinx (47). Furthermore, long-term stress increased C. muridarum load and pathology (48, 49), so tunnel handling as in our study (50, 51) may have reduced stress compared to standard tail-handling experiments, though we did not perform physiological or behavioral measures to support this.

C. muridarum *in vitro* passage may reduce hydrosalpinx formation and severity (52). Our passage of C. muridarum in LLC cells, as opposed to commonly used HeLa, McCoy, or Hec1B cells (43, 47, 53), might thus have also influenced pathogenicity. Though we observed peak shedding and course of decline to latency similar to that previously reported for progesterone-assisted vaginal inoculation of BALB/c mice with Hec1B-propagated C. muridarum (53), we cannot rule out an effect of propagation parameters. Notably, we observed no genital tissue-associated pathology associated with coinfection. We observed background oviduct dilation, cervix inflammatory cells, and especially, vaginal inflammatory cells, which may be due to the frequent vaginal manipulations (inoculation, swabbing), intraperitoneal antibiotic administration or the effects of antibiotics on vaginal normal flora. However, our finding that coinfection of latent genital C. muridarum-infected mice with N. gonorrhoeae did not result in increased vaginal PMNs, compared to N. gonorrhoeae single infection, in contrast to observations for acute genital C. muridarum/N. gonorrhoeae-coinfected mice versus N. gonorrhoeae singly infected mice (27), may be explained by a lack of increased vaginal N. gonorrhoeae infection/shedding in our setting.

Finally, our observed trend toward *reduced* vaginal PMNs in latent genital C. muridarum/N. gonorrhoeae-coinfected versus singly infected mice (N. gonorrhoeae > C. muridarum+N. gonorrhoeae > C. muridarum, both in vaginal tissues and vaginal smears representing luminal PMNs), contrasts previous *acute* genital C. muridarum/N. gonorrhoeae findings (27). This may support the recent finding that chlamydial PMN infection renders PMNs less responsive to N. gonorrhoeae (10). Chlamydial protease-like activity factor (CPAF) cleaved human PMN N-formyl peptide receptor 2 (FPR2), “paralyzing” the PMNs, and also inhibited murine PMNs (which express a FRP2 homologue) (10, 54). Thus, our findings may indicate that latent genital chlamydial infection may inhibit murine PMNs *in vivo*. This finding was not associated with modulation of C. muridarum or N. gonorrhoeae vaginal shedding/recovery or measures of tissue pathology in our study. However, we may speculate that a similar phenomenon in humans might reduce overall N. gonorrhoeae symptoms, perhaps hindering or delaying diagnosis or treatment and representing a source of potential transmission.

In summary, we show (i) that N. gonorrhoeae coinfection does not reactivate C. muridarum vaginal shedding from a state of C. muridarum genital latency; (ii) that latent chlamydial genital infection, unlike acute chlamydial genital infection as previously reported (27), does not increase recovery of viable vaginal N. gonorrhoeae; and (iii) that latent genital C. muridarum infection may limit subsequent N. gonorrhoeae-dependent vaginal PMN induction, consistent with chlamydial inhibition of normal PMN function against N. gonorrhoeae (10). Our findings expand on previous findings to suggest differing effects of acute versus latent chlamydial genital infection on subsequent N. gonorrhoeae genital infection in mice. Continued evaluation of Chlamydia/N. gonorrhoeae interaction *in vivo*, including how such coinfections may impact the host, is important to inform our continued efforts toward the prevention and/or treatment of chlamydia and gonorrhea.

## MATERIALS AND METHODS

### Host cells and media.

LLC-MK2 cells (LLC; rhesus monkey kidney cell line; provided by IZSLER, Brescia, Italy) were cultivated as previously described (28).

### *C. muridarum* propagation and inoculum preparation.

C. muridarum Weiss strain (obtained from Kyle Ramsey, Midwestern University) was propagated in LLC cells, crude stocks were prepared, and IFU/mL were determined as previously described (28, 55, 56).

### Mycoplasma testing.

DNA extracted from cells and chlamydial stock was tested using a mycoplasma detection kit (VenorGeM OneStep; MB Minerva Biolabs, Germany) for conventional PCR according to the manufacturer’s instructions. Cells and chlamydial stock used in this study were *Mycoplasma* negative.

### *N. gonorrhoeae* propagation and inoculum preparation.

N. gonorrhoeae strain FA1090 (endocervical isolate, originally isolated in 1983 from a probable disseminated infection) (57) was provided by Magnus Unemo, School of Medical Sciences, Orebro University, and cultivated on commercially available chocolate agar (chocolate agar with Vitox; Thermo Fisher Scientific), as previously described (28). For inocula, N. gonorrhoeae colonies (18 to 24 h) were collected with a sterile nylon swab and suspended in sterile phosphate-buffered saline (PBS [pH 7.2], 1-L tablets; Canvax Biotech, Córdoba, Spain) to a McFarland density of approximately 2.0 to 3.0 (DEN-1B densitometer; Grant Instruments, Cambridgeshire, UK; 0.5-4.0 McFarland Standard Set, Pro Lab Diagnostics, Richmond Hill, Ontario, Canada). The suspended N. gonorrhoeae was then passed through a sterile 1.2-μm-pore size filter to remove aggregates, as previously described (33), and diluted with sterile PBS to a McFarland density of approximately 0.62 to 0.67, which we determined by trial to represent CFU/mL of ~10^8^ CFU/mL. Inocula CFU/mL ranged from (1.7 to 2.2) × 10^6^, as determined by dilution of the filtered suspensions in sterile 0.05% saponin (Sigma-Aldrich/MilliporeSigma)/PBS cultured on chocolate agar for 18 to 24 h with bacterial colonies counted on a stereo microscope (M3; Wild Heerbrugg AG, Heerbrugg, Switzerland).

### Coinfection protocol.

Female BALB/c mice, 6 weeks of age, were purchased from Janvier Labs (Le Genest-Saint-Isle, France), housed five mice per cage with 12-h light/dark cycle, regulated temperature/humidity, with *ad libitum* access to standard mouse feed and water, and allowed to acclimate for 12 days with the inclusion of a tunnel for both enrichment and mouse handling as refinement to reduce stress (50, 51). The experimental design is shown in Fig. 1; the day of the experiment is noted as “day 0” to “day 36.” The study comprised 90 mice evaluated in three independent experiments carried out over 9 months between November 2020 and July 2021; (see File S1). Mice were divided into four groups: mice coinfected with C. muridarum and N. gonorrhoeae (C. muridarum+N. gonorrhoeae), mice infected with either pathogen alone (C. muridarum or N. gonorrhoeae), and mice mock infected with SPG (sucrose phosphate glutamate)/PBS alone as a control (UN). Mice inoculated with C. muridarum (the C. muridarum and C. muridarum+N. gonorrhoeae groups) were vaginally inoculated for three consecutive days with 10^6^ IFU of C. muridarum in 10 μL of SPG (sucrose phosphate glutamate buffer, 218 mM sucrose; Sigma-Aldrich/Millipore Sigma); 3.76 mM KH_2_PO_4_ (Sigma-Aldrich/Millipore Sigma), 7.1 mM K_2_HPO_4_ (Sigma-Aldrich/Millipore Sigma), and 5 mM GlutaMAX (Gibco, Thermo Fisher Scientific) on days 0, 1, and 2 to increase the likelihood that mice were in the diestrus stage of the reproductive cycle, which is critical for a robust chlamydial infection (30). Mice not inoculated with C. muridarum (the UN and N. gonorrhoeae groups) were vaginally similarly inoculated with SPG alone.

On experiment day 25, 23 days after chlamydial infection, during chlamydial latency, N. gonorrhoeae infection was carried out essentially as described previously (27, 33). Vaginal smear slides were prepared from all mice and stained with Hematek Stain Pak 4481 modified Wright stain (Siemens AG; Munich, Germany). Mice with predominantly vaginal neutrophils and nucleated epithelial cells, rather than cornified epithelial cells (as determined by cell morphology [33]), were considered to be in diestrus, while mice with few cells, visible bacteria and mucus were considered to be in anestrus. Mice in diestrus or anestrus were treated with subcutaneous injections of Premarin (0.5 mg), a conjugated estrogen, once per day mornings; (Sigma) on days 23, 25, and 27 to promote gonococcal infection and prolonged colonization. Intraperitoneal injections of vancomycin hydrochloride (0.6 mg) and streptomycin sulfate (2.4 mg) were given to all mice twice daily, mornings and afternoons, for 4 days (from days 23 to 27; on day 23, the vancomycin/streptomycin was administered only once in the afternoon) to control growth of commensal flora, caused by Premarin treatment. Moreover, from days 28 to 36, 5 g/L streptomycin sulfate dissolved in autoclaved tap water was provided *ad libitum* to all groups to continue to help control commensal flora overgrowth. Mice not in diestrus or anestrus on day 23 were sacrificed that day.

On day 25, ~4 h after the second dose of Premarin, mice were inoculated vaginally with either 20 μL of PBS (UN and C. muridarum groups) or with N. gonorrhoeae in 20 μL of PBS [range, (1.7 to 2.2) × 10^6^ CFU; N. gonorrhoeae and C. muridarum+N. gonorrhoeae groups]. Vaginal swabs, collected every 2 to 3 days, until the end of experiment, were used to determine first vaginal chlamydial shedding of live EBs (indicative of active C. muridarum infection) and chlamydial latency (lack of vaginal shedding of live EBs) by titer assay (days 4 to 24). After N. gonorrhoeae infection (days 27 to 36), both C. muridarum shedding and recovery of viable N. gonorrhoeae from vaginal swabs were determined by titration. At day 36, all mice were sacrificed, necropsy was performed, and tissue samples were collected and stored for histopathology and molecular analysis. In addition, at sacrifice, rectal swabs were collected for C. muridarum shedding determination, and vaginal smear slides were prepared for PMN analysis. Researchers were not blinded to mouse procedures or sample preparations, due to technical requirements. However, all microscopic analyses were carried out in a blinded manner, with the exception of pathological and immunohistochemistry analyses of tissues.

### Sample collection.

**(i) Vaginal and rectal swabs and vaginal smears.** Mice were vaginally or rectally swabbed with a PBS-soaked swab (Ultra Mini Flocked Swab, Copan 516CS01; Copan Italia, Brescia, Italy) inserted into the vaginal canal or rectum and rotated gently. Swabs were collected into 2-mL tubes containing 1 mL of sterile SPG and three sterile glass beads. Swab tubes were vortexed for 10 s and processed for recovery of viable N. gonorrhoeae (vaginal swabs only, see below) was carried out prior to snap-freezing on dry ice and storage at –80°C. Vaginal smears were collected by inserting a PBS-moistened swab into the vagina, rotating gently as for vaginal swabs, and smearing the collected material on standard glass microscopy slides.

**(ii) Necropsy, tissue sampling, and pathology scoring.** Necropsy included observation of gross genital tract macroscopic findings. The genital tract (ovary, oviduct, uterus, cervix, and vagina) and the intestinal tract (duodenum, jejunum, ileum, cecum, colon, and rectum) were collected and stored in 4% buffered formaldehyde for 24 h, followed by embedding in paraffin according to routine procedure. From each formalin-fixed and paraffin-embedded (FFPE) block, 2-μm sections were cut and stained with hematoxylin and eosin (H&E), followed by microscopic evaluation of complete longitudinal sections by a board-certified pathologist.

Histologic evaluation focused on the oviduct, cervix, and vagina. For the oviduct assessment, available cross sections on the slide were counted and the number of dilated versus total number of cross sections was calculated. In addition, the presence of inflammation (yes/no) was recorded, and the type of inflammatory infiltrate was described in a qualitative manner. For the cervix and the vagina, the stage of epithelial differentiation was recorded, and the presence of neutrophils was assessed semiquantitatively (luminal versus epithelial, mild, moderate, and severe).

### Quantification of viable chlamydial shedding by titration and immunofluorescence microscopy.

Quantification of vaginal or rectal viable C. muridarum shedding was determined by titration in LLC cells. Briefly, after overnight LLC culture, triplicate serial dilutions of thawed, vortexed swab samples were used to infect LLC as previously described (28). Inocula were replaced with growth medium supplemented with cycloheximide (Sigma) at a 1.5-μg/mL final concentration and antibiotics (amphotericin B [Gibco] at a 1.3-μg/mL final concentration, vancomycin hydrochloride [Sigma] at a 100-μg/mL final concentration, and gentamicin [Gibco] at a 10-μg/mL final concentration), and plates were incubated for 24 h at 37°C and 5% CO_2_. The cells were then fixed, and C. muridarum inclusions were immunostained as previously reported (28) Chlamydial inclusions were counted at a ×100 to ×200 magnification, and IFU/swab calculation was performed (limit of detection was 5 IFU/swab, at 200 μL of total swab volume assayed in the least diluted triplicate titration wells; Eclipse TiU; Nikon Instruments, Inc., Melville, NY). Vaginal swabs across all experiments for all groups (UN and N. gonorrhoeae) not C. muridarum inoculated were assayed as described, except 300 μL of total swab volume was assayed undiluted in triplicate wells (100 μL/well), to confirm the absence of C. muridarum cross-contamination.

### Quantification of recovery of viable *N. gonorrhoeae* by titration.

Swab samples vortexed for 5 to 10 s were added, in triplicate, to 0.05% saponin-PBS, in 96-well U-bottom plates, and 1:3 serial dilutions (eight dilutions) were performed; then, 5 μL of each dilution well and three 34-μL replicates of undiluted sample were plated to commercial selective agar plates (*Neisseria* Selective Medium PLUS; Thermo Scientific) with antibiotic selection (vancomycin, colistin, amphotericin B, and trimethoprim), followed by incubation for 24 to 48 h at 37°C and 5% CO_2_. N. gonorrhoeae colonies were counted on a stereomicroscope (M3; Wild Heerbrugg AG), and N. gonorrhoeae shedding (CFU/swab) was calculated (the limit of detection was 10 CFU/swab, at 100 μL of undiluted sample assayed). Vaginal swabs for all groups across all experiments (UN and C. muridarum) not N. gonorrhoeae inoculated were assayed as described, except that 50 μL of total swab volume was assayed to confirm the absence of N. gonorrhoeae cross-contamination.

### DNA extraction and *Chlamydiaceae* real-time PCR.

DNA extracted from 150 μL of vaginal swab or rectal swab samples, using the QIAamp DNA minikit (Qiagen, Hilden, Germany) according to the manufacturer’s recommendations, was eluted in a 50-μL final volume. DNA extracted from tissue samples (20-μm sections of FFPE blocks as previously described [58]) was eluted in a 50-μL final volume. C. muridarum positivity (expressed in units of genome copies/swab) was determined by quantitative real-time PCR (qPCR) based on the Chlamydiaceae family-specific 23S rRNA gene, as previously described (58, 59). All samples were tested in duplicate at 7.5 μL of total swab volume assayed as 2.5 μL of DNA per qPCR (limit of detection, ~10^2^ genome copies), and a cycle threshold (*C_T_*) value of <38 was considered to be positive for C. muridarum and used to calculate chlamydial genome copies/rectal swab, while vaginal swab and tissue samples were scored only as negative or positive; a positive control comprising a 7-fold *C. abortus* DNA dilution series and a negative control (water instead of template DNA) were included in each run (60).

### CM and *N. gonorrhoeae* detection by immunohistochemistry.

Complete longitudinal genital tract sections of all mice (*n* = 89) were immunolabeled for C. muridarum and N. gonorrhoeae. Intestinal tract tissue sections (consisting of one cross section each from the duodenum, jejunum, ileum, cecum, colon, and rectum) from the C. muridarum and C. muridarum+N. gonorrhoeae groups (*n* = 49) were immunolabeled for C. muridarum, and those from the N. gonorrhoeae and C. muridarum+N. gonorrhoeae groups (*n* = 59) were immunolabeled for N. gonorrhoeae. Primary antibodies included a Chlamydiaceae family-specific rabbit polyclonal antibody LPS/MOMP antibody (Cygnus Technologies, Inc., Southport, NC) at a 1:1,000 dilution for C. muridarum antigen detection and a rabbit anti-N. gonorrhoeae polyclonal antibody (Abnova, Taipeh, Tawain) at a 1:1,000 dilution for N. gonorrhoeae. Antigen retrieval consisted of pressure cooking (98°C) in citrate buffer (Dako/Agilent, Santa Clara, CA) for 20 min for Chlamydia and treatment with FastEnzyme (Zytomed Systems GmbH, Bargteheide, Germany) for 10 min at room temperature for *Neisseria*, respectively. After incubation with the primary antibodies for 1 h at room temperature, the endogenous peroxidase activity (Dako Agilent) was inhibited for 10 min at room temperature. Detection was performed with Envision + System HRP Rabbit (Dako Agilent) for a 30-min incubation at room temperature, using the substrate 3-amino-9-ethylcarbazole (AEC)-peroxidase with a hematoxylin counterstain. For the positive controls, lung tissue from a C. pneumoniae-infected mouse (Chlamydia control, kindly provided by Bernhard Kaltenboeck) and a cell pellet array, including N. gonorrhoeae-infected LLC-MK2 and HeLa cells, was used.

### PMN semiquantification.

Vaginal smear slides prepared at the end of the experiment (day 36) were stained to semiquantify the PMNs. Briefly, smears were stained with Hematek stain, evaluated (as described above), and categorized as having (i) no/rare PMNs (consistent with estrus as induced by estradiol/estrogen) versus (ii) few/more PMNs (consistent with PMN influx into the vaginal lumen).

### Statistical analysis.

Sample size for the primary analyses of C. muridarum and N. gonorrhoeae vaginal shedding was estimated based on simulated power analysis under design of two-way analysis of variance with a repeated-measures model. A final sample size of *n* = 14 mice per group (except for the mock-treated group, which was not directly compared, and considering mice dropped out due to estrous stage) was determined to ensure the probability of 90% that the expected power is at least 0.80. Experimental units were completely randomized to experimental groups in each of the independent experiments.

The significance of any observed differences in viable C. muridarum and N. gonorrhoeae vaginal shedding between experimental groups was evaluated using a linear mixed-effect model, taking into account repeated measures over time of the same mouse and excluding any mice for which viable C. muridarum shedding or recovery of viable N. gonorrhoeae could not be detected in any of the 12 vaginal swabs collected. Analysis was performed on log_10_-transformed IFU/swab and CFU/swab data, with values of 1 IFU or CFU below the limit of detection (i.e., 4 and 9, respectively) assigned to individual swab samples with no C. muridarum or N. gonorrhoeae detected.

The significance of any observed differences was evaluated for five pathology outcomes (oviduct dilation ratio [dilated/total], oviduct dilation score [none, mild, moderate, or severe], oviduct inflammation [yes versus no], as well as cervical and vaginal infiltration with PMNs graded from 0 to 3 [none, mild, moderate, or severe]). Early-sacrifice mice were evaluated as two groups: mock infected (the UN and N. gonorrhoeae groups) or C. muridarum infected (the C. muridarum and C. muridarum+N. gonorrhoeae groups). Late-sacrifice mice were evaluated as four groups: UN, C. muridarum, N. gonorrhoeae, and C. muridarum+N. gonorrhoeae. Mice with tissue sections missing were excluded from pathology analyses. For the first pathology outcome, the oviduct dilation ratio, which is a continuous variable, we applied the *t* test. For the other four pathology outcomes which are either ordinal or categorical variables, we applied the Fisher exact test.

The significance of any observed differences in live C. muridarum rectal shedding or C. muridarum genome copies/swab between experimental groups were evaluated using the *t* test; analysis was performed on log_10_-transformed IFU/swab and, with values of 1 IFU below the limit of detection (i.e., 4) assigned to individual swab samples with no C. muridarum detected. The proportion of mice C. muridarum rectal swabs determined to be positive by titer and by Chlamydiaceae qPCR, respectively, was compared between the C. muridarum and C. muridarum+N. gonorrhoeae groups, and the proportion of mice N. gonorrhoeae IHC positive in the genital tract was compared between the N. gonorrhoeae and C. muridarum+N. gonorrhoeae groups; for these comparisons we applied the Fisher exact test.

Statistical analysis was performed using R statistical software (https://www.R-project.org/).

### Animal use.

Unless otherwise stated (as in File S3), animal experiments were conducted in the Laboratory Animal Services Center (LASC) at University of Zurich (BSL-2) and previously approved by Cantonal Veterinarian’s Office of Zurich (license 018/2020). Refinements were made to minimize animal stress.

### Data availability.

The original contributions presented are included within the article and in the supplemental material. Further inquiries can be directed to the corresponding author.

## References

[1] World Health Organization. 2021. Global progress report on HIV, viral hepatitis, and sexually transmitted infections 2021: accountability for the global health sector strategies, 2016–2021, p 1689–1699, vol 53. World Health Organization, Geneva, Switzerland.

[2] Department of Health and Human Services. 2020. Sexually transmitted infection national strategic plan for the United States: 2021–2025. Disease Control Division (TB/Leprosy Section 12), Department of Health and Human Services, Washington, DC.

[3] Wind CM, Schim van der Loeff MF, Unemo M, Schuurman R, van Dam AP, de Vries HJC. 2016. Time to clearance of *Chlamydia trachomatis* RNA and DNA after treatment in patients coinfected with *Neisseria gonorrhoeae*: a prospective cohort study. BMC Infect Dis 16:554. doi:10.1186/s12879-016-1878-3. 27724878 PMC5057251

[4] Batteiger BE, Tu W, Ofner S, Van Der Pol B, Stothard DR, Orr DP, Katz BP, Fortenberry JD. 2010. Repeated *Chlamydia trachomatis* genital infections in adolescent women. J Infect Dis 201:42–51. doi:10.1086/648734. 19929379 PMC2791188

[5] Unemo M, Bradshaw CS, Hocking JS, de Vries HJC, Francis SC, Mabey D, Marrazzo JM, Sonder GJB, Schwebke JR, Hoornenborg E, Peeling RW, Philip SS, Low N, Fairley CK. 2017. Sexually transmitted infections: challenges ahead. Lancet Infect Dis 17:e235–e279. doi:10.1016/S1473-3099(17)30310-9. 28701272

[6] Fatima F, Kumar S, Das A. 2022. Vaccines against sexually transmitted infections: an update. Clin Exp Dermatol 47:1454–1463. doi:10.1111/ced.15223. 35426443

[7] Elwell C, Mirrashidi K, Engel J. 2016. Chlamydia cell biology and pathogenesis. Nat Rev Microbiol 14:385–400. doi:10.1038/nrmicro.2016.30. 27108705 PMC4886739

[8] Hafner LM, Wilson DP, Timms P. 2014. Development status and future prospects for a vaccine against *Chlamydia trachomatis* infection. Vaccine 32:1563–1571. doi:10.1016/j.vaccine.2013.08.020. 23973245

[9] Redgrove KA, McLaughlin EA. 2014. The role of the immune response in *Chlamydia trachomatis* infection of the male genital tract: a double-edged sword. Front Immunol 5. doi:10.3389/fimmu.2014.00534. PMC4209867 25386180

[10] Rajeeve K, Das S, Prusty BK, Rudel T. 2018. *Chlamydia trachomatis* paralyses neutrophils to evade the host innate immune response. Nat Microbiol 3:824–835. doi:10.1038/s41564-018-0182-y. 29946164

[11] Quillin SJ, Seifert HS. 2018. *Neisseria gonorrhoeae* host adaptation and pathogenesis. Nat Rev Microbiol 16:226–240. doi:10.1038/nrmicro.2017.169. 29430011 PMC6329377

[12] Criss AK, Seifert HS. 2012. A bacterial siren song: intimate interactions between *Neisseria* and neutrophils. Nat Rev Microbiol 10:178–190. doi:10.1038/nrmicro2713. 22290508 PMC3569855

[13] Darville T. 2021. Pelvic inflammatory disease due to *Neisseria gonorrhoeae* and *Chlamydia trachomatis*: immune evasion mechanisms and pathogenic disease pathways. J Infect Dis 224:S39–S46. doi:10.1093/infdis/jiab031. 34396413 PMC8365118

[14] Dicker LW, Mosure DJ, Berman SM, Levine WC. 2003. Gonorrhea prevalence and coinfection with chlamydia in women in the United States, 2000. Sex Transm Dis 30:472–476. doi:10.1097/00007435-200305000-00016. 12916141

[15] Stupiansky NW, Van Der Pol B, Williams JA, Weaver B, Taylor SE, Fortenberry JD. 2011. The natural history of incident gonococcal infection in adolescent women. Sex Transm Dis 38:750–754. doi:10.1097/OLQ.0b013e31820ff9a4. 21317686

[16] Hillis SD, Nakashima A, Marchbanks PA, Addiss DG, Davis JP. 1994. Risk factors for recurrent *Chlamydia trachomatis* infections in women. Am J Obstet Gynecol 170:801–806. doi:10.1016/s0002-9378(94)70286-1. 8141205

[17] Haggerty CL, Gottlieb SL, Taylor BD, Low N, Xu F, Ness RB. 2010. Risk of sequelae after *Chlamydia trachomatis* genital infection in women. J Infect Dis 201:134–155. doi:10.1086/652395. 20470050

[18] Oriel JD, Ridgway GL. 1982. Epidemiology of chlamydial infection of the human genital tract: evidence for the existence of latent infections. Eur J Clin Microbiol 1:69–75. doi:10.1007/BF02014194. 7173175

[19] Batteiger BE, Fraiz J, Newhall VW, Katz BP, Jones RB. 1989. Association of recurrent chlamydial infection with gonorrhea. J Infect Dis 159:661–669. doi:10.1093/infdis/159.4.661. 2926160

[20] Lin JS, Donegan SP, Heeren TC, Greenberg M, Flaherty EE, Haivanis R, Su XH, Dean D, Newhall WJ, Knapp JS, Sarafian SK, Rice RJ, Morse SA, Rice PA. 1998. Transmission of *Chlamydia trachomatis* and *Neisseria gonorrhoeae* among men with urethritis and their female sex partners. J Infect Dis 178:1707–1712. doi:10.1086/314485. 9815223

[21] Barron AL, White HJ, Rank RG, Soloff BL, Moses EB. 1981. A new animal model for the study of *Chlamydia trachomatis* genital infections: infection of mice with the agent of mouse pneumonitis. J Infect Dis 143:63–66. doi:10.1093/infdis/143.1.63. 7217713

[22] Darville T, Andrews CW, Laffoon KK, Shymasani W, Kishen LR, Rank RG. 1997. Mouse strain-dependent variation in the course and outcome of chlamydial genital tract infection is associated with differences in host response. Infect Immun 65:3065–3073. doi:10.1128/iai.65.8.3065-3073.1997. 9234755 PMC175432

[23] Jerse AE. 1999. Experimental gonococcal genital tract infection and opacity protein expression in estradiol-treated mice. Infect Immun 67:5699–5708. doi:10.1128/IAI.67.11.5699-5708.1999. 10531218 PMC96944

[24] Packiam M, Wu H, Veit SJ, Mavrogiorgos N, Jerse AE, Ingalls RR. 2012. Protective role of Toll-like receptor 4 in experimental gonococcal infection of female mice. Mucosal Immunol 5:19–29. doi:10.1038/mi.2011.38. 21937985 PMC3240729

[25] Dockterman J, Coers J. 2021. Immunopathogenesis of genital *Chlamydia* infection: insights from mouse models. Pathog Dis 79:ftab012. doi:10.1093/femspd/ftab012. 33538819 PMC8189015

[26] Jerse AE, Wu H, Packiam M, Vonck RA, Begum AA, Garvin LE. 2011. Estradiol-treated female mice as surrogate hosts for *Neisseria gonorrhoeae* genital tract infections. Front Microbiol 2:1–13. doi:10.3389/fmicb.2011.00107. 21747807 PMC3129519

[27] Vonck RA, Darville T, O’Connell CM, Jerse AE. 2011. Chlamydial infection increases gonococcal colonization in a novel murine coinfection model. Infect Immun 79:1566–1577. doi:10.1128/IAI.01155-10. 21245268 PMC3067530

[28] Onorini D, Borel N, Schoborg RV, Leonard CA. 2022. *Neisseria gonorrhoeae* limits *Chlamydia trachomatis* inclusion development and infectivity in a novel *in vitro* coinfection model. Front Cell Infect Microbiol 12:1–15. doi:10.3389/fcimb.2022.911818. PMC9300984 35873141

[29] Imtiaz MT, Schripsema JH, Sigar IM, Ramsey KH. 2006. Outcome of urogenital infection with *Chlamydia muridarum* in CD-14 gene knockout mice. BMC Infect Dis 6:144. doi:10.1186/1471-2334-6-144. 16995947 PMC1590040

[30] Rank RG. 1994. Animal models for urogenital infections. Methods Enzymol 235:83–93. doi:10.1016/0076-6879(94)35133-3. 8057939

[31] Perry LL, Hughes S. 1999. Chlamydial colonization of multiple mucosae following infection by any mucosal route. Infect Immun 67:3686–3689. doi:10.1128/IAI.67.7.3686-3689.1999. 10377161 PMC116566

[32] Rank RG, Yeruva L. 2014. Hidden in plain sight: chlamydial gastrointestinal infection and its relevance to persistence in human genital infection. Infect Immun 82:1362–1371. doi:10.1128/IAI.01244-13. 24421044 PMC3993372

[33] Raterman EL, Jerse AE. 2019. Female mouse model of *Neisseria gonorrhoeae* infection. Methods Mol Biol 1997:413–429. doi:10.1007/978-1-4939-9496-0_24. 31119637

[34] Connolly KL, Pilligua-Lucas M, Gomez C, Costenoble-Caherty AC, Soc A, Underwood K, Macintyre AN, Sempowski GD, Jerse AE. 2021. Preclinical testing of vaccines and therapeutics for gonorrhea in female mouse models of lower and upper reproductive tract infection. J Infect Dis 224:S152–S160. doi:10.1093/infdis/jiab211. 34396408 PMC8365121

[35] Russell AN, Zheng X, O’Connell CM, Taylor BD, Wiesenfeld HC, Hillier SL, Zhong W, Darville T. 2016. Analysis of factors driving incident and ascending infection and the role of serum antibody in *Chlamydia trachomatis* genital tract infection. J Infect Dis 213:523–531. doi:10.1093/infdis/jiv438. 26347571 PMC4721908

[36] Nsuami M, Cammarata CL, Brooks BN, Taylor SN, Martin DH. 2004. Chlamydia and gonorrhea co-occurrence in a high school population. Sex Transm Dis 31:424–427. doi:10.1097/01.olq.0000130535.96576.d3. 15215698

[37] Ramsey KH, Sigar IM, Schripsema JH, Denman CJ, Bowlin AK, Myers GAS, Rank RG. 2009. Strain and virulence diversity in the mouse pathogen *Chlamydia muridarum*. Infect Immun 77:3284–3293. doi:10.1128/IAI.00147-09. 19470744 PMC2715693

[38] Cotter TW, Miranpuri GS, Ramsey KH, Poulsen CE, Byrne GI. 1997. Reactivation of chlamydial genital tract infection in mice. Infect Immun 65:2067–2073. doi:10.1128/iai.65.6.2067-2073.1997. 9169733 PMC175285

[39] Wang L, Zhang Q, Zhang T, Zhang Y, Zhu C, Sun X, Zhang N, Xue M, Zhong G. 2016. The *Chlamydia muridarum* organisms fail to auto-inoculate the mouse genital tract after colonization in the gastrointestinal tract for 70 days. PLoS One 11:e0155880. doi:10.1371/journal.pone.0155880. 27192556 PMC4871562

[40] Wang L, Zhu C, Zhang T, Tian Q, Zhang N, Morrison S. 2018. Nonpathogenic colonization with chlamydia in the gastrointestinal tract as oral vaccination for inducing transmucosal protection. Infect Immun 86. doi:10.1128/IAI.00630-17. PMC5778366 29133348

[41] Tian Q, Zhou Z, Wang L, Abu-Khdeir A-MH, Huo Z, Sun X, et al. 2020. Gastrointestinal coinfection promotes chlamydial pathogenicity in the genital tract. Infect Immun 88:17–20. doi:10.1128/IAI.00905-19. PMC7093119 31988173

[42] Panzetta ME, Valdivia RH, Saka HA. 2018. *Chlamydia* persistence: a survival strategy to evade antimicrobial effects *in vitro* and *in vivo*. Front Microbiol 9:3101. doi:10.3389/fmicb.2018.03101. 30619180 PMC6299033

[43] Chen J, Zhang H, Zhou Z, Yang Z, Ding Y, Zhou Z, Zhong E, Arulanandam B, Baseman J, Zhong G. 2014. Chlamydial induction of hydrosalpinx in 11 strains of mice reveals multiple host mechanisms for preventing upper genital tract pathology. PLoS One 9:e95076. doi:10.1371/journal.pone.0095076. 24736397 PMC3988139

[44] Yu H, Lin H, Xie L, Tang L, Chen J, Zhou Z, et al. 2019. *Chlamydia muridarum* induces pathology in the female upper genital tract via distinct mechanisms. Infect Immun 87:1–11. doi:10.1128/IAI.00145-19. PMC6652751 31085708

[45] Andrew DW, Cochrane M, Schripsema JH, Ramsey KH, Dando SJ, O’Meara CP, et al. 2013. The duration of *Chlamydia muridarum* genital tract infection and associated chronic pathological changes are reduced in IL-17 knockout mice but protection is not increased further by immunization. PLoS One 8:1–14. doi:10.1371/journal.pone.0076664. PMC3779189 24073293

[46] de la Maza LM, Pal S, Khamesipour A, Peterson EM. 1994. Intravaginal inoculation of mice with the *Chlamydia trachomatis* mouse pneumonitis biovar results in infertility. Infect Immun 62:2094–2097. doi:10.1128/iai.62.5.2094-2097.1994. 8168974 PMC186471

[47] Maxion HK, Liu W, Chang M-HH, Kelly KA. 2004. The infecting dose of *Chlamydia muridarum* modulates the innate immune response and ascending infection. Infect Immun 72:6330–6340. doi:10.1128/IAI.72.11.6330-6340.2004. 15501762 PMC523032

[48] Belay T, Woart A. 2013. Cold-induced stress increases the intensity of *Chlamydia* genital infection in mice. J Microbiol Immunol Infect 46:330–337. doi:10.1016/j.jmii.2012.06.002. 22789437

[49] Belay T, Woart A, Graffeo V. 2017. Effect of cold water-induced stress on immune response, pathology and fertility in mice during *Chlamydia muridarum* genital infection. Pathog Dis 75. doi:10.1093/femspd/ftx045. PMC5808652 28431099

[50] Hurst JL, West RS. 2010. Taming anxiety in laboratory mice. Nat Methods 7:825–826. doi:10.1038/nmeth.1500. 20835246

[51] Gouveia K, Hurst JL. 2013. Reducing mouse anxiety during handling: effect of experience with handling tunnels. PLoS One 8. doi:10.1371/journal.pone.0066401. PMC3688777 23840458

[52] Chen C, Zhou Z, Conrad T, Yang Z, Dai J, Li Z, et al. 2015. *In vitro* passage selects for *Chlamydia muridarum* with enhanced infectivity in cultured cells but attenuated pathogenicity in mouse upper genital tract. Infect Immun 83:1881–1892. doi:10.1128/IAI.03158-14. 25712926 PMC4399068

[53] Phillips Campbell R, Kintner J, Whittimore J, Schoborg RV. 2012. *Chlamydia muridarum* enters a viable but non-infectious state in amoxicillin-treated BALB/c mice. Microbes Infect 14:1177–1185. doi:10.1016/j.micinf.2012.07.017. 22943883 PMC3654801

[54] Sogawa Y, Ohyama T, Maeda H, Hirahara K. 2011. Inhibition of neutrophil migration in mice by mouse formyl peptide receptors 1 and 2 dual agonist: indication of cross-desensitization *in vivo*. Immunology 132:441–450. doi:10.1111/j.1365-2567.2010.03367.x. 21039475 PMC3044910

[55] Marangoni A, Bergamini C, Fato R, Cavallini C, Donati M, Nardini P, et al. 2014. Infection of human monocytes by *Chlamydia pneumoniae* and *Chlamydia trachomatis*: an *in vitro* comparative study. BMC Res Notes 7:230. doi:10.1186/1756-0500-7-230. 24721461 PMC3984436

[56] Onorini D, Donati M, Marti H, Biondi R, Levi A, Nufer L, et al. 2019. The influence of centrifugation and incubation temperatures on various veterinary and human chlamydial species. Vet Microbiol 233:11–20. doi:10.1016/j.vetmic.2019.04.012. 31176395

[57] Cohen MS, Cannon JG, Jerse AE, Charniga LM, Isbey SF, Whicker LG. 1994. Human experimentation with *Neisseria gonorrhoeae*: rationale, methods, and implications for the biology of infection and vaccine development. J Infect Dis 169:532–537. doi:10.1093/infdis/169.3.532. 8158024

[58] Borel N, Marti H, Pospischil A, Pesch T, Prähauser B, Wunderlin S, et al. 2018. Chlamydiae in human intestinal biopsy samples. Pathog Dis 76. doi:10.1093/femspd/fty081. PMC6276272 30445531

[59] Blumer S, Greub G, Waldvogel A, Hässig M, Thoma R, Tschuor A, Pospischil A, Borel N. 2011. *Waddlia*, *Parachlamydia*, and *Chlamydiaceae* in bovine abortion. Vet Microbiol 152:385–393. doi:10.1016/j.vetmic.2011.05.024. 21658867

[60] Hoffmann K, Schott F, Donati M, Di Francesco A, Hässig M, Wanninger S, Sidler X, Borel N. 2015. Prevalence of chlamydial infections in fattening pigs and their influencing factors. PLoS One 10:e0143576. doi:10.1371/journal.pone.0143576. 26619187 PMC4664257

