# Supplementary material for: Neisseria gonorrhoeae Coinfection during Chlamydia muridarum Genital Latency Does Not Modulate Murine Vaginal Bacterial Shedding
Source: Microbiol Spectr. 2023 Apr 11;11(3):e04500-22. doi: 10.1128/spectrum.04500-22 (PMC10269798; doi:10.1128/spectrum.04500-22)
Supplement: Supplemental file 1 — Supplemental material. Download spectrum.04500-22-s0001.pdf, PDF file, 0.6 MB [file spectrum.04500-22-s0001.pdf]

SUPPLEMENTAL FILE 1. Study Design.

Expt	Group	Cage	Mouse	Early Sac	Status
1	NG	1	1	No	OK
1	NG	1	2	No	OK
1	NG	1	3	No	OK
1	NG	1	4	Yes	ND
1	NG	1	5	No	OK
1	CM+NG	1	1	No	OK
1	CM+NG	1	2	No	NNG
1	CM+NG	1	3	No	OK
1	CM+NG	1	4	Yes	ND
1	CM+NG	1	5	No	OK

…..

Expt	Group	Cage	Mouse	Early Sac	Status
2	UN	1	1	No	OK
2	UN	1	2	Yes	ND
2	UN	1	3	No	OK
2	UN	1	4	Yes	ND
2	UN	1	5	Yes	ND
2	UN	2	6	Yes	ND
2	UN	2	7	Yes	ND
2	UN	2	8	Yes	ND
2	UN	2	9	No	OK
2	UN	2	10	No	OK
2	CM	1	1	No	OK
2	CM	1	2	No	OK
2	CM	1	3	Yes	ND
2	CM	1	4	No	OK
2	CM	1	5	No	OK
2	CM	2	6	No	OK
2	CM	2	7	No	OK
2	CM	2	8	Yes	ND
2	CM	2	9	No	OK
2	CM	2	10	No	OK
2	NG	1	1	No	NNG
2	NG	1	2	No	OK
2	NG	1	3	No	OK
2	NG	1	4	Yes	ND
2	NG	1	5	Yes	ND
2	NG	2	6	No	OK
2	NG	2	7	Yes	ND
2	NG	2	8	Yes	ND
2	NG	2	9	No	NNG
2	NG	2	10	No	OK
2	CM+NG	1	1	No	OK
2	CM+NG	1	2	Yes	ND
2	CM+NG	1	3	NA	SpD
2	CM+NG	1	4	Yes	ND
2	CM+NG	1	5	No	OK
2	CM+NG	2	6	Yes	ND
2	CM+NG	2	7	No	OK
2	CM+NG	2	8	Yes	ND
2	CM+NG	2	9	No	NNG
2	CM+NG	2	10	No	OK

…..

Expt	Group	Cage	Mouse	Early Sac	Status
3	CM	1	1	Yes	ND
3	CM	1	2	No	OK
3	CM	1	3	No	OK
3	CM	1	4	No	OK
3	CM	1	5	No	OK
3	CM	2	6	Yes	ND
3	CM	2	7	Yes	ND
3	CM	2	8	No	OK
3	CM	2	9	No	OK
3	CM	2	10	No	OK
3	NG	1	1	No	NNG
3	NG	1	2	No	OK
3	NG	1	3	No	OK
3	NG	1	4	No	NNG
3	NG	1	5	No	OK
3	NG	2	6	No	OK
3	NG	2	7	No	OK
3	NG	2	8	No	NNG
3	NG	2	9	Yes	ND
3	NG	2	10	No	OK
3	NG	3	11	No	OK
3	NG	3	12	No	OK
3	NG	3	13	No	OK
3	NG	3	14	No	NNG
3	NG	3	15	No	OK
3	CM+NG	1	1	No	NNG
3	CM+NG	1	2	No	OK
3	CM+NG	1	3	Yes	ND
3	CM+NG	1	4	No	NNG
3	CM+NG	1	5	No	OK
3	CM+NG	2	6	No	OK
3	CM+NG	2	7	No	OK
3	CM+NG	2	8	No	OK
3	CM+NG	2	9	Yes	ND
3	CM+NG	2	10	No	OK
3	CM+NG	3	11	No	OK
3	CM+NG	3	12	No	NCM
3	CM+NG	3	13	No	OK
3	CM+NG	3	14	No	OK
3	CM+NG	3	15	Yes	ND

Key: CM (*Chlamydia muridarum*) = infected with CM alone; NG (*Neisseria gonorrhoeae*) = infected with NG alone; CM+NG = co-infected with CM and NG; UN = uninfected (control); Early Sac = sacrificed on Day 23; ND = not in Diestrus/Anestrus on Day 23; OK = in Diestrus/Anestrus and successfully CM and/or NG infected (included in vaginal bacterial shedding analyses); NCM = in Diestrus/Anestrus on Day 23, but CM infection not detected (excluded from vaginal bacterial shedding analyses); NNG = in Diestrus/Anestrus on Day 23, but NG infection not detected (excluded from vaginal bacterial shedding analyses); SpD = died spontaneously on Day 1, excluded from further analyses; NA = not applicable.

SUPPLEMENTAL FILE 2. Health Score Sheet

**Mouse Health Score Sheet**		
**Parameter**	**Description**	**Score**
**Appearance**<br>Assessed in open cage	Bright eyes; groomed fur; active, twitching whiskers; erect ears; moist, pink nose	**0**
	Dull, ungroomed/soiled fur; clumped whiskers or flattened ears	**2**
	Grimace as indicated by orbital tightening, nose bulge or cheek bulge	**2**
**Natural Behavior**<br>Assessed in open cage<br>(observe for one minute)	Active, easy movement; interactive in cage and with cagemates; looks at observer	**0**
	Slight decrease in activity; less interactive; disregards observer	**1**
	Pronounced decrease in activity; isolated from cagemates; fails to move if cage is disturbed; hyperactive	**2**
**Provoked Behavior**<br>Gentle nudge in cage	Quickly moves away	**0**
	Slow to move away or exaggerated response	**1**
	Moves away, but only after extended pause	**2**
**Body Condition**<br>Assessed on cage top	Well-conditioned: dorsal pelvis/vertebrae not prominent, dorsal pelvis palpable with slight pressure	**0**
	Underconditioned: vertebrae clearly visible, dorsal pelvis easily palpable	**2**
**External Vaginal Appearance**<br>Assessed on cage top	No redness, swelling or discharge	**0**
	Discharge in the absence of redness and swelling	**1**
	Redness and/or swelling, with/without discharge	**2**
**Injection Site Appearance**<br>Assessed on cage top	No redness or swelling	**0**
	Redness and/or swelling	**2**

Analysis:

**Sum of Score Points**	**Severity**	**Action**
0		
1	1	Euthanize when lasting 3 days
Score points of 1, totaling 2-3	1	Euthanize when lasting 2 days
ANY score point of 2	2	Euthanize immediately

References:

- Langford, Dale J., et al. "Coding of facial expressions of pain in the laboratory mouse." *Nature methods* 7.6 (2010): 447.
- Paster, Eden V., Kimberly A. Villines, and Debra L. Hickman. "Endpoints for mouse abdominal tumor models: refinement of current criteria." *Comparative medicine* 59.3 (2009): 234-241.
- Ullman-Culleré, Mollie H., and Charmaine J. Foltz. "Body condition scoring: a rapid and accurate method for assessing health status in mice." *Comparative Medicine* 49.3 (1999): 319-323.

SUPPLEMENTAL FILE 3. Cyclophosphamide immune suppression preliminary experiments.

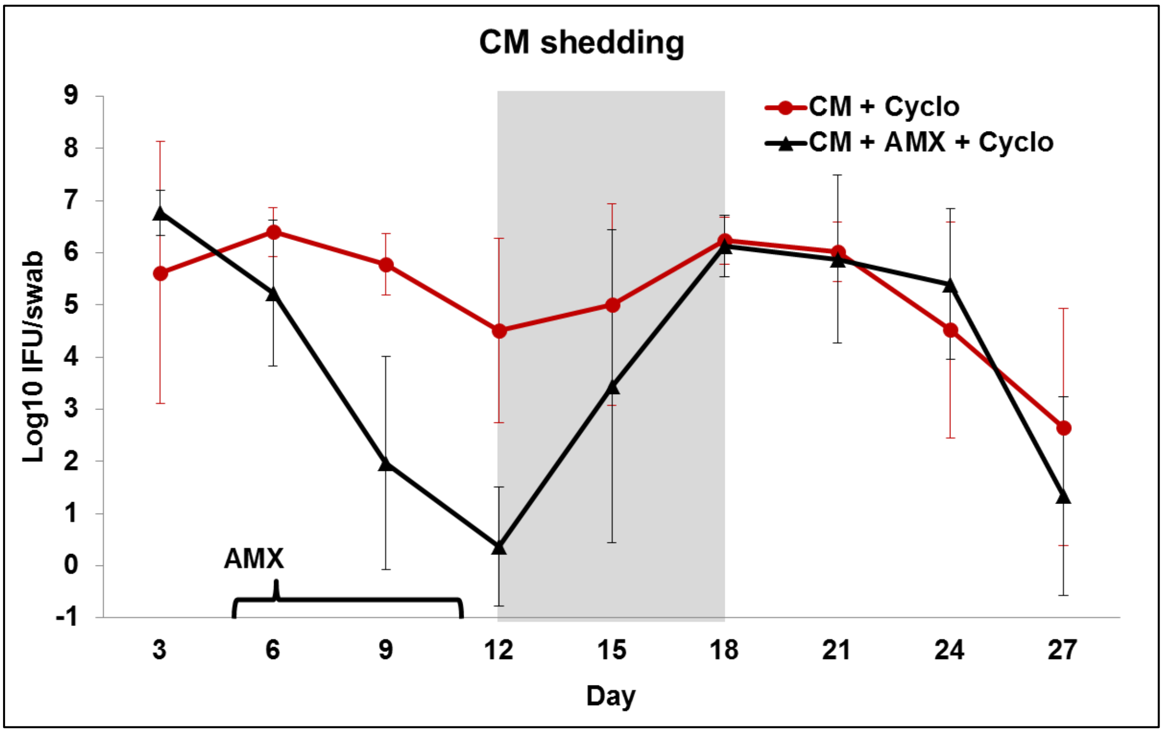

Viable *Chlamydia muridarum* (CM) vaginal shedding[1]									
	**Day 3**	**Day 6**	**Day 9**	**Day 12**	**Day 15**	**Day 18**	**Day 21**	**Day 24**	**Day 27**
**CM + Cyclo**	17/20	20/20	20/20	18/20	18/20	20/20	20/20	17/20	12/20
**CM + AMX + Cyclo**	20/20	19/20	9/20	2/20	12/20	20/20	19/20	19/20	7/20
[1]Positive/total mice									

Supplemental File 3 Cylophosphamide (Cyclo) immune suppression increases *Chlamydia muridarum* (CM) viable vaginal shedding **(upper panel)** and proportion of mice with detectable shedding **(lower panel)** in mice allowed to progress naturally to latency (CM + Cyclo; n = 20) and in mice with amoxicillin (AMX)-induced chlamydial persistence (CM + AMX + Cyclo; n = 20). Quantification of viable CM shedding was determined by infecting HeLa 229 cells and the results are expressed as mean log_{10} inclusion forming units (IFU)/vaginal swab +/- standard deviation. Shaded area **(upper panel)** indicates days of Cyclo treatment (Days 12-18); mice treated with AMX were treated from Days 5-11 as indicated by the bracket. Data are from two independent experiments of n = 10 mice per group.

Supplemental File 3 Materials and Methods

Chlamydia, cells, and animals

C. muridarum Weiss (CM) was obtained from Dr. Kyle Ramsey and propagated in the Hec1B human endometrial cell line (ATCC# HTB-113) using bead culture (Guseva et al., 2007). Chlamydial stocks were titered in BM1.11 murine oviduct epithelial cells, generously provided by Dr. Ray Johnson (Johnson, 2004), to determine total inclusion forming units (IFU)/mL. Vaginal swabs were titered on HeLa 229 monolayers (ATCC# CCL2.1). Six-week-old female BALB/c mice (Harlan, USA) were

acclimated to the East Tennessee State University Quillen College of Medicine Division of Laboratory Animal Resources (DLAR) animal facilities for two weeks, after which they were treated with 2.5 mg Depo-provera (Depo) by subcutaneous injection to synchronize their menstrual cycles and increase infection efficiency. Mice were aged for two weeks prior to Depo treatment, as we observed significant mortality from cyclophosphamide (Cylo) treatment in younger animals. Notably, allowing mice to age one additional week reduced Cyclo-related mortality from 50% to 0%, respectively. One week after Depo treatment, ten mice per group were vaginally infected with 10^6 IFU of CM in 10 μl of 2SPG or an equal volume of 2SPG alone (0.2 M sucrose, 20 mM phosphate buffer, and 5 mM L-glutamine). Mice were sacrificed 27 days post infection (dpi). This study was carried out in accordance with the recommendations in the Guide for the Care and Use of Laboratory Animals of the National Institutes of Health. The protocol was approved by the University Committee on Animal Care of East Tennessee State University (Protocol Number: P120201). All efforts were made to minimize pain and discomfort.

AMX and Cyclo treatments in vivo

Mice were treated with 2 mg/kg AMX in sterile water twice daily by gavage at 5-7 dpi, a regimen previously shown to induce persistence (Phillips Campbell et al., 2012). Control mice were gavaged with sterile water. For immune suppression, mice received a loading dose (200 mg/kg) of Cyclo by intraperitoneal injection on 12 dpi. Maintenance doses (40 mg/kg) were continued by once daily intraperitoneal injection on 12-18 dpi, a regimen previously shown to suppress both humoral and cell-mediated immunity in mice (Otterness & Chang, 1976).

Quantification of chlamydial shedding

Mice were vaginally swabbed every third day and swabs were snap frozen in 2 ml tubes containing 500 μl of 2SPG and three 3 mm glass beads. Samples were later thawed, vortexed, briefly sonicated and sample dilutions were used to infect HeLa 229 cells plated at 10^5 cells per well on glass coverslips in 24 well plates. Cultures were centrifuged at 1100 g for 1 h and refed with antibiotic/antifungal medium. Chlamydial inclusions were allowed to develop for 24 h before being fixed and permeablized. Inclusions were enumerated using Pathfinder anti-chlamydial LPS fluorescent stain for visualization.

Supplemental File 3 References

Guseva NV, Dessus-Babus S, Moore CG, Whittimore JD, Wyrick PB. Differences in *Chlamydia trachomatis* serovar E growth rate in polarized endometrial and endocervical epithelial cells grown in three-dimensional culture. *Infect Immun*. 2007;75(2):553-564. doi:10.1128/IAI.01517-06

Johnson RM. Murine oviduct epithelial cell cytokine responses to *Chlamydia muridarum* infection include interleukin-12-p70 secretion. *Infect Immun*. 2004;72(7):3951-3960. doi:10.1128/IAI.72.7.3951-3960.2004

Phillips Campbell R, Kintner J, Whittimore J, Schoborg RV. *Chlamydia muridarum* enters a viable but non-infectious state in amoxicillin-treated BALB/c mice. Microbes Infect. 2012;14(13):1177-1185. doi:10.1016/j.micinf.2012.07.017

Otterness IG, Chang YH. Comparative study of cyclophosphamide, 6-mercaptopurine, azathiopurine and methotrexate. Relative effects on the humoral and the cellular immune response in the mouse. *Clin Exp Immuno*l. 1976;26(2):346-354.

SUPPLEMENTAL TABLE 1 Vaginal Swabs; CM titer and *Chlamydia* (CM) qPCR[1]

Days 4-21			Days 24-36		
	**CM titer**	**CM qPCR**		**CM titer**	**CM qPCR**
**Mock-infected**	0/35	0/35	**NG**	0/20	0/20
**CM-infected**	14/31 (45.2%)	14/31 (45.2%)	**CM+NG**	0/20	0/20

[1] Positive/total (percent) vaginal swabs

SUPPLEMENTAL TABLE 2 Genital Tissue *Chlamydia* (CM) qPCR[1]

Early Sacrifice		Late Sacrifice	
**Mock-infected**	0/12	**UN**	0/4
		**CM**	0/7
**CM-infected**	2/12 (16.7%)	**NG**	0/10
		**CM+NG**	0/9

[1] Positive/total (percent) mice

SUPPLEMENTAL TABLE 3 Vaginal Tissue *N. gonorrhoeae* Immunohistochemistry[1]

Early Sacrifice		Late Sacrifice	
**Mock-infected**	0/11	**UN**	0/2
		**CM**	0/8
**CM-infected**	0/7	**NG**	9/22 (40.9%)
		**CM+NG**	4/20 (20%)

[1] Positive/total (percent) mice

SUPPLEMENTAL TABLE 4 Intestinal Tissue *Chlamydia* (CM) qPCR[1]

Early Sacrifice		Late Sacrifice	
**Mock-infected**	0/12	**UN**	0/4
		**CM**	3/10 (30%)
**CM-infected**	12/22 (54.5%)	**NG**	0/10
		**CM+NG**	1/10 (10%)

[1] Positive/total (percent) mice

SUPPLEMENTAL TABLE 5 Oviduct Pathology, Dilation and Inflammation[1]

Early Sacrifice			Late Sacrifice		
	**Dilation**	**Inflammation**		**Dilation**	**Inflammation**
**Mock-infected**	2/12 (16.7%)	0/12	**UN**	0/4	0/4
			**CM**	6/15 (40%)	4/15 (26.7%)
**CM-infected**	3/12 (25%)	3/12 (25%)	**NG**	4/24 (16.7%)	1/24 (4.2%)
			**CM+NG**	6/20 (30%)	3/20 (15%)

[1] Positive/total (percent) mice

SUPPLEMENTAL TABLE 6 Cervical Inflammation[1]

Early Sacrifice		Late Sacrifice	
**Mock-infected**	5/12 (41.7%)	**UN**	0/3
		**CM**	5/15 (33.3%)
**CM-infected**	4/13 (30.8%)	**NG**	6/22 (27.3%)
		**CM+NG**	8/20 (40%)

[1] Positive/total (percent) mice

SUPPLEMENTAL TABLE 7 Vaginal Inflammation[1]

Early Sacrifice		Late Sacrifice	
**Mock-infected**	9/12 (75%)	**UN**	2/3 (66.7%)
		**CM**	6/15 (40%)
**CM-infected**	6/13 (46.2%)	**NG**	15/22 (68.2%)
		**CM+NG**	10/19 (52.6%)

[1] Positive/total (percent) mice
